# Health as a rich people’s game through the lens of work and income

**DOI:** 10.1186/s12889-025-23320-z

**Published:** 2025-05-31

**Authors:** Wen-Jui Han, Marc A. Scott, Nora Tang

**Affiliations:** 1 New York University, 1 Washington Square North, New York, NY 10003 USA; 2 New York University, Kimball Hall 246 Greene Street Floor 3, New York, NY 10003 United States of America

**Keywords:** Health, Physical function, Mental function, CES-D depressive symptoms, Life course perspective, NLSY79, Nonstandard work schedules, Sequence analysis, Social determinants of health

## Abstract

**Background:**

The growing reliance on precarious employment —work that is uncertain, insecure, and unstable —has transformed work for many from a resource to a vulnerability for health and well-being.

**Methods:**

Using longitudinal data from the NLSY79 cohort (*n* ≈ 6,666) in the United States, we focused on two social determinants of health (SDOH), work and family income. We examine work schedule and income patterns between ages 22 and 49, explicitly building upon the life course lens to answer how such patterns before age 50 may shape future health outcomes at age 50. We used sequence analysis to categorize work and family income trajectories and multivariate regression to examine the relationship between work and family income trajectories on health at age 50.

**Results:**

Our sequence analysis reveals four diverse work clusters ranging from stable standard daytime hours to volatile work patterns (e.g., not working, working evening or night hours, or variable hours) and four family income clusters ranging from upward mobility to persistent low-income patterns. Our multivariate regression suggests a strong income gradient in health, which plays a critical role in cushioning the otherwise adverse effects on health from volatile work patterns. In contrast, limited and volatile income exacerbates the negative effects of volatile work patterns on health. These adverse associations were particularly pronounced for females.

**Conclusions:**

Taken together, our results demonstrate a strong income gradient in health that may be moderated by diverse work patterns, with significant implications for how work and income as SDOH factors play critical roles in shaping intergenerational poverty and inequality.

**Clinical trial number:**

Not applicable.

**Supplementary Information:**

The online version contains supplementary material available at 10.1186/s12889-025-23320-z.

## Background

This paper focuses on two social determinants of health (SDOH) — work and income —using a life course lens to examine their relationship with physical and mental health at age 50. Employment is an important avenue for obtaining social and economic resources, allowing individuals and families to afford a decent standard of living [1, 2]. In addition to earnings, jobs may offer critical social protections such as health insurance and paid leave, which are resources conducive to nurturing health and well-being [1]. However, the growing reliance on work that is uncertain, insecure, and unstable, so-called precarious employment [3–5], has transformed work for many from a resource to a vulnerability for our health and well-being, partly due to inadequate material compensation and precarious working conditions [3, 6]. Precarious employment includes features such as low wages; unpredictable or nonstandard hours; (involuntary) part-time hours; and temporary, on-call, or irregular shifts —often without social protections (e.g., health insurance, paid sick days, family leave). These workers have always been among the most vulnerable groups in the workforce; the COVID-19 pandemic further eroded and worsened this brutal reality for these workers, who were hit the hardest, with millions losing their income and jobs [7–9]. We focus on the interwoven nature of work and income to shed light on the intersectionality of social determinants of health on our health from a long-term perspective.

We focus our investigation of precarity on jobs requiring nonstandard work schedules such as evenings, nights, rotating shifts, or irregular hours because these jobs tend to offer low wages, part-time work, and few to no social protections [10–11]. The fact that low wages are the reality for many workers with nonstandard work schedules challenges our ability to disentangle the work and income effects associated with health. Scholarship has suggested that one mechanism linking nonstandard work schedules and adverse health is inadequate resources gained from these jobs, raising concerns about feedback mechanisms whereby work, income, and health interact over time, possibly leading to reverse causality [3, 10]. For example, jobs requiring nonstandard work schedules may bring inadequate earnings, and inadequate earnings may hinder one’s ability to change job trajectories, resulting in work and individual income reinforcing each other as health evolves and interacts. Inadequate family income would have similar effects due to the high correlation between nonstandard work schedules, low wages, and low family income [12–13]. Our study addresses these issues using a life course perspective with rigorous temporal sequences between these variables, tapping into two distinct yet interrelated associations with health: cumulative work and family income effects, which we explore individually and jointly.

The distinct role of individual versus family resources on health is worth noting. Whereas individual income is a critical indicator of an individual’s economic situation and potential health risks, individual income may not fully capture the economic resources available to individuals, especially through contributions from other household members when living with others. Individuals might also choose specific work arrangements based on family income context, such as choosing not to work if they perceive economic resources through other family members might meet their needs, including those related to health. In addition, focusing on individual income does not allow us to examine the income effects of those who were not working and therefore did not have wage/salary data. Hence, family income more accurately reflects a family’s ability to meet its members’ financial needs, including those related to health [14]; this choice also allows this analysis to be comparable with studies examining the links between (family) income and health [14]. For brevity, we use “income” hereafter to denote “family income” unless otherwise specified.

We are mindful that nonstandard work schedules are only one aspect of precarious employment, and our findings should not be extrapolated to the health consequences of precarious employment in general. We also acknowledge that not all nonstandard work schedules are economically disadvantageous. Some might voluntarily choose such schedules based on individual preferences or other considerations. For example, in the 2004 Current Population Survey, a small share of workers (≈ 6%), most of whom were young workers without children, indicated they chose night shifts for higher pay and an easier commute (authors’ calculation).

### Work (schedules), income, and health

Work can directly affect health, such as due to unsafe working conditions (e.g., chemical hazards, excessive temperatures) [15–16]. In addition, the characteristics of work may indirectly shape health. Examples include uncertain, unstable, or unpredictable hours; nontraditional hours, such as nights or weekends; low or poverty wages; (involuntary) schedule changes; frequently shifting between working and joblessness (insecure employment); and part-time hours. We build upon a line of studies showing that unemployment and nonstandard schedules may produce physical strain and emotional stress, on top of bringing inadequate resources [3, 10, 15, 17, 18]. Extant empirical evidence has shown that unemployment is associated with feelings of worry, anxiety, depression [19–20], and physical pain [16], and that people who were unemployed suffered more from stress-related illnesses such as stroke, heart disease, and arthritis [19, 21]. In addition, one study found that people who frequently changed jobs were more likely to smoke, consume more alcohol, and exercise less, no matter their gender, age, or socioeconomic status (SES) [22]. Similarly, a growing body of work has shown, both in the U.S. and globally, that a nonstandard work schedule, when compelled involuntarily, is less conducive to our health [15, 23–25]. Nonstandard work schedules may interrupt sleep routines [26] or lead to a lack of physical exercise [27]. Importantly, research has suggested that the high likelihood of engaging in risky health behaviors among workers with nonstandard schedules might be due to job stress [27]. Such workers were more likely to sleep fewer than 6 h a day or to have poor sleep quality, smoke more, and consume 2 + alcoholic drinks per day [27]. Insufficient sleep, the most common risky behavior among workers with precarious jobs [24], increases the odds of depression [28], high blood pressure, heart disease, and stroke [29]. Unstable or irregular hours combined with low wages may also create work-related physical and psychological stress [15, 27], resulting in chronic conditions such as type 2 diabetes or heart disease [24, 27, 30] and poor mental health such as depression, anxiety, or distress [24, 27]. Lastly, working evening or night hours tends to impact family routines (e.g., family dinners), which, in turn, can trigger cognitive appraisal of stress and a conflict between individuals’ work and family spheres [31].

Income is, of course, closely related to work and hence affects health through work. But income also shapes health by enabling (or hindering) access to health care and resources for a healthy lifestyle. Therefore, low income may induce stress concerning workers’ ability to support themselves and their families financially and through worry about their ability to weather health issues [14, 32–33]. Insufficient wages and, consequently, low family income, cause both economic and emotional stress, inducing and increasing risky health behaviors (e.g., smoking and drinking) [34–35]. Recent health statistics on US adults show an income gradient in health risks (chronic conditions, fair or poor health, health limitations in usual activities). For example, 24% of adults with an annual family income under $35,000 reported limitations due to one or more chronic conditions compared to 15%, 12%, 10%, and 7% of those with family income increasing in $15,000 intervals (top coded at $100,000 or more) [36]. The most recent Surgeon General’s report on tobacco health disparities has reaffirmed the disproportionate burden carried by people with limited resources, who were twice as likely to smoke than their counterparts; in 2020, 20% of those making under $35,000 a year reported being current cigarette smokers compared to 6% of those making $100,000 or more per year [37]. Smoking causes about 80–90% of lung cancer deaths in the U.S., and low SES is associated with higher lung cancer risk [34]. These statistics illustrate a strong association between stress-induced coping behaviors, economic deprivation, and poor health. While we focus on income affecting health, we are mindful that health may limit work opportunities and the kind of work individuals engage in, which, in turn, could affect income. Hence, a bidirectional relationship between income and health is likely but beyond the scope of this paper.

Importantly, work as both a resource and a vulnerability generates life trajectories that produce considerable differences in access to work-related resources and achieving social status, producing health disparities. Inadequate resources, including low wages and low family income, limit individuals’ ability to access health-promoting resources (e.g., health insurance, healthy food) and compound the adverse impacts of precarious work characteristics. Together and cumulatively, work and low income may become daily stressors, creating cumulative effects of chronic stress that weigh heavily on physical and mental health through the persistent activation of the body’s stress response (allostatic load). Chronic stress can disrupt the biological regulation of stress response systems, including metabolism and the immune system [38], creating a vicious cycle of unhealthy stress responses, causing inflammation and other physical health issues [14, 39].

The extant research has also suggested that some groups may carry disproportionate burdens from work, including women, racial-ethnic minorities, and those with low educational achievement [10, 40–42]. For example, recent research has attributed health disparities between men and women to structural sexism at multiple levels [40]. Gender attitudes and behaviors can lead to inequality at the individual level, marital dyad level (e.g., power and relations between husbands and wives), and system level (e.g., opportunities and constraints structured by gender differences including workplace practices such as earnings, job tasks, work hours) [40]. Structural sexism on all levels has disproportionately disadvantaged women, increasing women’s exposure to (role-related) stress and diminishing their access to material resources, adversely affecting health and well-being [40, 43]. Structural sexism can manifest in family work decisions: Studies have found that women are likely to choose nonstandard work schedules if such an arrangement helps satisfy family and work demands [44–45]. Regardless of work schedules, women are also more likely than men to hold low-paying and often insecure service jobs [46]. Studies have found that family demands impact women’s careers more than men’s [47]; family responsibilities often trigger occupational sorting into lower-paying jobs, enabling women to reconcile work and care by working part-time or non-day hours [48, 49]. Juggling family and work demands, in combination with low-paying and insecure jobs, can have a heavy toll on women’s health, a manifestation of structural sexism [24].

### The importance of a life course lens

Our health is an ever-evolving product of our daily behaviors and experiences shaped by our surrounding environments. While snapshots of health can be helpful, a life course approach captures the complexities of dynamic changes and transitions in relation to work and income and is essential for devising effective social policies and programs [50–51]. This approach considers the role of work and income in shaping cumulative processes and the critical transitions between different work and income statuses. Using longitudinal data, we explore the association between one’s work and income throughout adulthood and subsequent health outcomes at age 50. We examine work schedule and income patterns between ages 22 and 49, explicitly building upon the principle that the past shapes the future to answer how such patterns *before age 50* may shape future health outcomes *at age 50*. We posit that the timing and sequencing of transitions may differentially affect one’s later health and explore this expectation in our life course analysis. Given the interconnected nature of work and income, we investigate health as a manifestation of the “joint forces” of these two critical SDOH factors, which produce both opportunities and resources, generating increasing disparities in resources (e.g., health, wealth) between those who have and those who have not.

### The present study

We focus on both work and family income patterns throughout one’s working life to examine health in middle adulthood, moving beyond point-in-time outcomes. We also take a longitudinal perspective to conceptualize the work–income interplay as a “process” that unfolds across individuals’ life courses during their prime working ages. In addition, instead of a unidirectional focus of either the effect of work on income or vice versa, we conceptualize work-income trajectories as interconnected. Using the National Longitudinal Survey of Youth, 1979 cohort (NLSY79), we examine the following questions: (1) What are the work (schedules) and income patterns between ages 22 and 49? (2) How are work and income patterns associated with health at age 50, individually and jointly? (3) How do these associations differ by gender, race-ethnicity, and education?

## Methods

### Data

This study used data from the NLSY79, conducted by the U.S. Department of Labor. The NLSY79 comprises a large nationally representative sample of 12,686 young men and women ages 14 to 22 who were first interviewed in 1979, with annual interviews conducted until 1994 and biennially thereafter. Of note, NLSY79 discontinued two oversamples: (1) military youth, discontinued in 1984 (*n* = 1,079 dropped and *n* = 201 retained), and (2) non-Black non-Hispanic disadvantaged youths, discontinued in 1990 (*n* = 1,643). This study excluded the oversampled military youths, who were likely to have different work patterns than non-military workers (*n* = 1,280). Excluding these two oversamples leaves 9,763 respondents as the starting point. Despite the NLSY79 team following up with the same participants for about 40 years, the response rates have been high, from 96% in the early years of the survey to about 77% in recent years [52]. The sample contains rich data, including histories of sociodemographic characteristics (e.g., education, marriage, income), annual work schedule histories, and physical and psychological well-being metrics. The NLSY79 thus remains the only longitudinal nationally representative sample that includes work schedule data over one’s working years.

We used age 22 instead of 18 as the starting point to document work-income patterns over one’s working years for two reasons. First, the cohort structure of the NLSY79 means we did not have data on age 18 for respondents aged 19–22 in 1979. Second, many respondents attended college between ages 18 and 22, during which time they were likely to work part-time with nonstandard hours; for them, age 22 is likely the starting point of establishing a career.

The analysis is focused on health at age 50. NLSY79 collected health outcomes through health modules at ages 40, 50, and 60. All participants had reached age 50 by 2020, but only a small proportion (30%) had reached age 60. We, thus, assessed health at age 50 to include most participants and to have the longest lens possible for determining work-income patterns and their associations with health.

### Participants

Respondents with missing information on the outcome variable at age 50 (*n* = 2,125 for SF-12 and *n* = 2,119 for CES-D) or with consistently missing information on work schedules and family income between ages 22 and 49 (*n* = 6) were excluded. Another 595 cases were dropped due to missing information on work schedules or family income that could not be imputed (details in Appendix A). In addition, about 5% of cases had missing information on sociodemographic characteristics (the missing rates ranged from less than 0.01% on education to 3.7% on respondents’ parents’ education; *n* = 371). After these exclusions, the final analyzed samples were 6,672 for the outcome variable of depressive symptoms and 6,666 for the physical and mental functions outcome variables (described below). The pattern of missing values on the three outcome measures suggests that the older age cohort (e.g., ages 19 or older in 1979) was more likely than the younger cohorts to have missing values on the outcome measures. This pattern may indicate a positive selection bias, with younger or healthier respondents more likely to remain in the longitudinal study. Following previous research, this study does not impute missing values for the outcome measures [53].

### Measures

*SF12 Physical and Mental Health*. The NLSY79 used the 12-Item Short-Form Health Survey (SF-12 v1) to assess self-reported mental and physical health as part of 50 + health modules for those who had turned 50 since their last interview (between interview years 2008 and 2016). The respondents were asked 12 questions about the past 4 weeks, including whether their health had caused them to limit moderate activities, whether pain had interfered with normal work, and their frequency of feeling downhearted or blue, with possible responses ranging from 1 (excellent) to 5 (poor). We used two global scores representing physical and mental functions created by the NLSY79 following the scoring established by Ware, Kosinski, and Keller [54]. Previous studies have shown that the SF-12 has good reliability (e.g., 0.89) and validity [55] and can detect active and recent depressive disorders [54]. NLSY79 standardized the scores to have a mean of 50 and a standard deviation of 10; a score of 50 corresponds to the U.S. average, and a one-point difference is one-tenth of a standard deviation [56]. Prior research has shown that the NLSY79 sample had a higher-than-average score on SF-12 mental function and just about the average score on SF-12 physical function [57]. The higher the score, the better the function is.

*Depressive Symptoms*. As part of age 50 + health modules, NLSY79 collected depressive symptoms using seven items of the Center for Epidemiologic Studies Depression Scale (CES-D) [58]. Respondents were asked how they felt during the week prior to the interview with prompts such as “I felt depressed,” “My sleep was restless,” and “I felt lonely” on a scale of 0 (rarely/none of the time/1 day) to 3 (most/all of the time/5–7 days). The NLSY79 created a total CES-D score by summing the responses of all seven questions, with a possible score of 0 to 21 and higher scores indicating more depressive symptoms. If one item was missing, the scale score was coded as missing. This short form of the CES-D has similar or higher reliability and validity compared to the original 20-item CES-D [59]. A score of 8 or greater was used to identify individuals with symptoms placing them at clinical risk of depression; prior studies have found this cutoff score to have acceptable specificity and modest sensitivity with the standard CES-D cutoff score of 16 [59].

*Work Schedule Patterns*. The NLSY79 surveyed participants about their work schedules every survey year. We followed the NLSY79 definition to identify five work statuses: “standard” if the work hours began at 6 a.m. or later and ended by 6 p.m.; “evenings” if the hours began at 2 p.m. or later and ended by midnight; “nights” if the hours began at 9 p.m. or later and ended by 8 a.m.; “variable” if the schedules were split shift, rotating shift, or irregular hours; and “not working” for those not working any job at the time of the interview.

*Family Income*. At each survey year, the NLSY79 collected income information from sources such as salaries/wages, business income, welfare assistance, alimony/child support, and other family members for the previous calendar year (i.e., income collected in 2000 referred to total family income made during calendar year 1999) and then created a composite figure of total net family income by combining income sources from household members. For our analyses, we converted the current dollar value to constant 2018 dollars.

*Sociodemographic Characteristics*. To address unobserved heterogeneity between participants and potential selection bias driving the links between work schedules, family income, and health [3, 14], we considered an extensive set of sociodemographic characteristics before or at age 22 as well as between ages 22 and 49. The analysis considers the following characteristics by age 22: age in 1979; sex in 1979 (female vs. male as the reference group; [ref.] hereafter); race-ethnicity in 1979 (non-Hispanic Black, Hispanic, Asian and others, and non-Hispanic White as the ref.); education by age 23 (less than high school, high school degree as the ref., some college, and college degree or higher); background information at age 14 including parental education (i.e., less than high school, high school as the ref., some college, or college degree or higher), not living with both biological parents, and residential location (suburban, rural, versus urban); any health issues limiting work by age 22; region of residence at age 22 (Northeast, Midwest, West, versus South); and early-life economic hardship if they had ever been in poverty and/or had received welfare assistance by age 22. Specifically, we considered participants to have ever lived in poverty by age 22 if their family income was at 100% of or under the federal poverty threshold during any of the survey years before or at age 22. Similarly, we considered participants to have ever received welfare benefits if they reported having ever received low-income cash assistance (Aid to Families with Dependent Children before 1996 and renamed TANF after 1996), Food Stamps (in-kind assistance, renamed the Supplemental Nutrition Assistance Program after 2008), or SSI (supplemental security income) before or at age 22. Our analysis also considered the following characteristics between ages 22 and 49: the number of marriages and children, average weekly working hours, and occupations. To assess average weekly working hours, we defined “only or mostly full-time hours” if participants worked 35 or more hours per week for at least half of the survey years between ages 22 and 49, “only or mostly part-time hours” if participants worked fewer than 35 h per week for at least half of the interview years between ages 22 and 49, or “mixed” if the share of full-time and part-time hours were equally distributed across the survey years between ages 22 and 49. Similarly, we identify participants to have “primarily professional/managerial” occupations if they worked at these occupations for at least half of the survey years between ages 22 and 49, with a similar approach to identify “mostly work at sales-related occupations,” “mostly work at service-related occupations,” and “mostly work at other occupations.” Participants who spent about an equal share of the years in different occupations were considered to have “mixed” occupations between ages 22 and 49.

### Empirical strategy

We began our analysis by addressing missing values associated with work and family income [60–61]. Next, we conducted separate sequence analyses [62–64] for work and family income. Appendix A provides details about this process [65–70]. Next, to examine the links between work-income patterns between ages 22 and 49 and health at age 50, we ran two separate ordinary least squares (OLS) models on SF-12 physical and mental functions and a logistic regression on the likelihood of being at clinical risk of depressive symptoms. All analyses considered all sociodemographic variables described in the Measures section. R software was used for clustering in the sequence analyses, with the TraMineR package to construct distances and for visualization [71]; Stata v.18 was used for the multiple regression analyses.

## Results

### Work and family income trajectories between ages 22 and 49

**Fig. 1:**
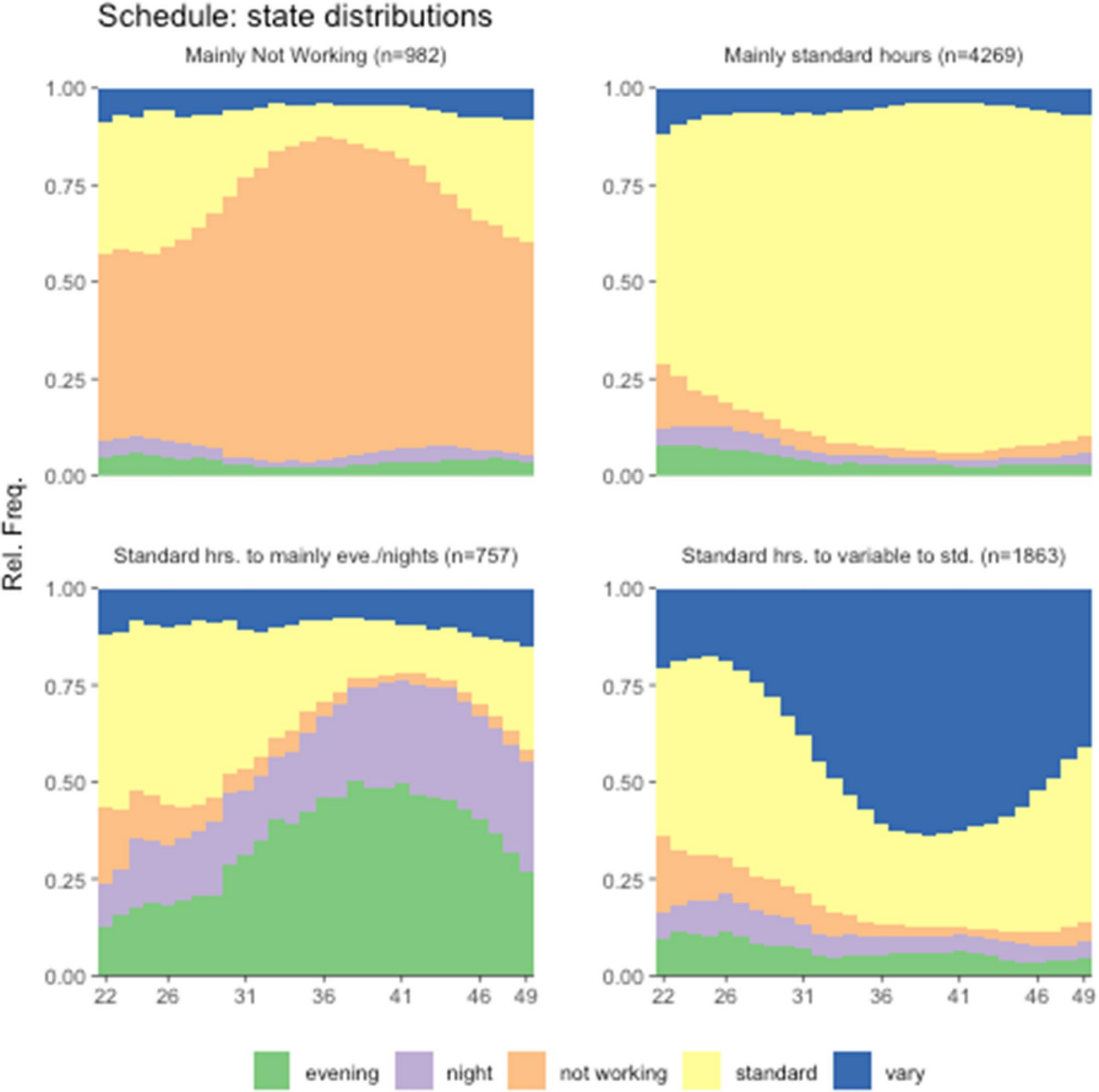
Sequence cluster solutions on work between ages 22 and 49, NLSY79 = 7,871

**Fig. 2:**
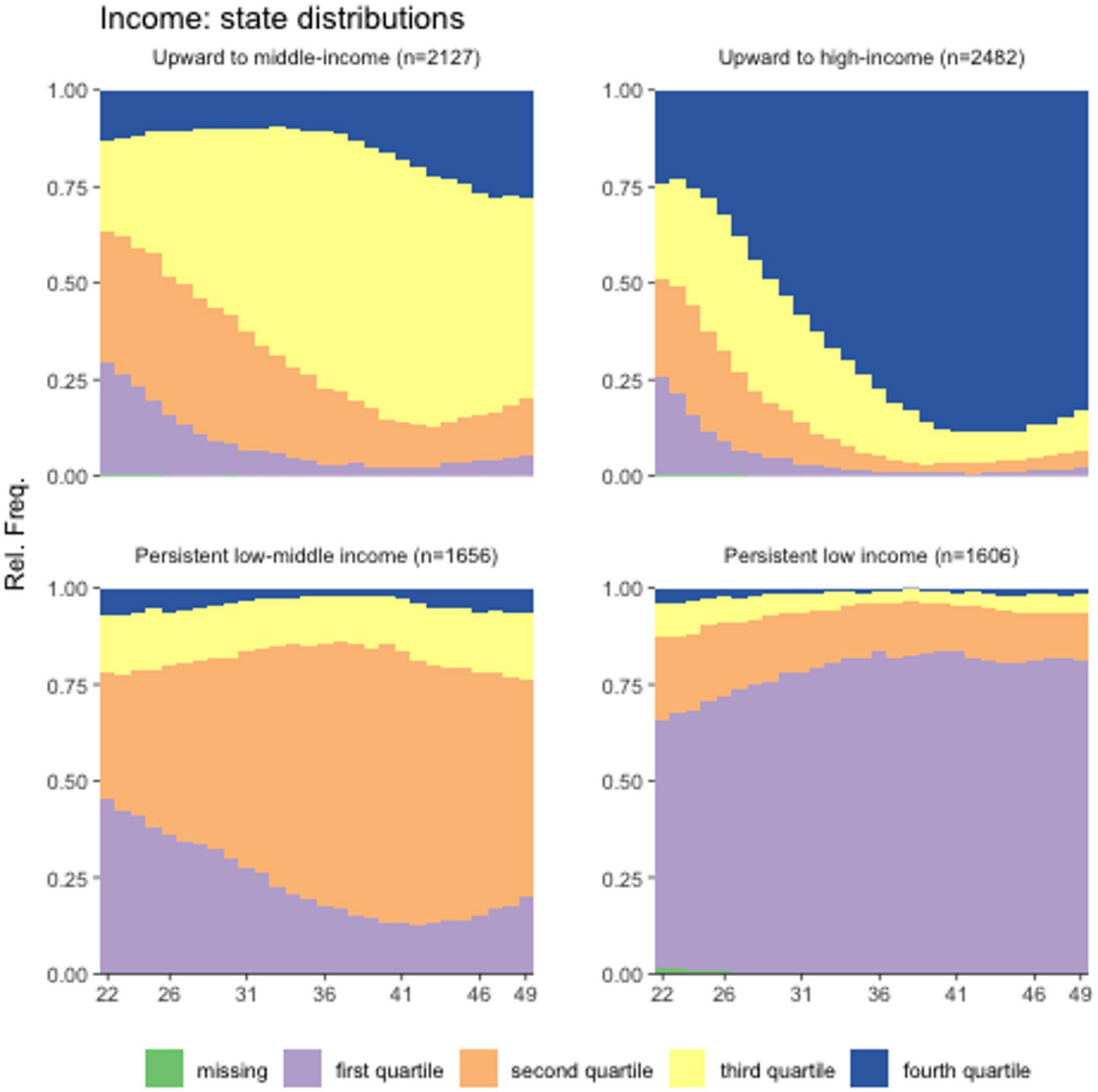
Sequence cluster solutions on family income between Ages 22 and 49, NLSY79 = 7,871

Figure 1 presents the four-cluster solution of the work-state sequence distributions (aggregated across individuals), and Fig. 2 shows the four-cluster solution of family income sequences between ages 22 and 49. Appendix Figs. 1 and 2 (see Additional files) present the sequence index plots of work and family income, respectively, detailing the transitions and changes by individuals over time. We label each cluster based on persistence or change over time. Appendix B provides more details about our clustering methodology. We are mindful that our labeling of each cluster reflects the dominant state within the cluster, but masks a great deal of diversity and dynamic changes in work and income over time (see Appendix Figs. 1 and 2 in Additional Files for a visualization of the dynamic changes and transitions). Hence, we note the dynamic and complex nature of transitions embedded in cluster solutions whenever possible.

We identify four general work patterns between ages 22 and 49 (see Fig. 1; Table 1). Cluster 1 comprises about 12% of the participants, with work patterns categorized as primarily not working (“mainly NW”) but with noticeable variations. Specifically, about half of the respondents began “not working”, but most moved in and out of different types of work schedules over time (including back to NW); about a third of the individuals began with standard work schedules but transitioned into not working by their 30s. Cluster 2 (about 54% of respondents) consists primarily of standard daytime schedules (“mainly ST”); this characterization holds particularly true after their mid-20s. For cluster 3 (10% of the respondents), about one-third of respondents had work schedule patterns that started with standard daytime schedules and then (slowly) transitioned to primarily evenings/nights (“ST to mainly evenings/nights”), so that in their 30s, these individuals had transitioned to evening-only or night-only schedules. Cluster 3 also has noticeable proportions of respondents engaged in variable hours throughout. Overall, individuals in cluster 3 did not switch back and forth between different work schedules, nor did they easily return to standard work schedules. Finally, cluster 4 (24% of the participants) may be characterized as starting with standard daytime schedules during their 20s, then transitioning to variable hours during their 30s, and then transitioning back to standard daytime hours toward their late 40s (“ST to variable to ST”), with a noticeable share of respondents either not working or working evenings/nights during their 20s.

**Table 1:** Work schedules and family income cluster patterns between ages 22 and 49, NLSY79

		Family	income		
Work Schedule	Upward to Middle-Income	Upward to High-Income	Persistently Low-Middle Income	Persistently Low Income	Row Total (%)
Mainly NW	127 (12.93%)	177 (18.02%)	114 (11.61%)	564 (35.12%)	982 (12.48%)
Mainly ST	1,255 (29.40%)	1,548 (36.26%)	927 (21.71%)	539 (12.63%)	4,269 (54.24%)
ST to mainly evenings/nights	227 (29.99%)	142 (18.76%)	198 (26.16%)	190 (25.10%)	757 (9.62%)
ST to variable to ST	518 (27.80%)	615 (33.01%)	417 (22.38%)	313 (16.80%)	1,863 (23.67%)
Column Total (%)	2,127 (27.02%)	2,482 (31.53%)	1,656 (21.04%)	1,606 (20.40%)	7,871 (100.00%)

Note. NW: not working; ST: standard daytime hours; row percentages are shown in cells

We also identify four predominant family income patterns (see Fig. 2; Table 1). Income cluster 1 (about 27% of respondents) has trajectories moving upward from lower to middle-income starting during their 30s (“upward to middle-income”), with a growing share, albeit small, moving to high income (4th quartile) toward their late 40s. Income cluster 2 (about 32% of respondents) starts equally divided across the four quartiles, with most moving to high-income (“upward to high-income”), achieving the top income quartile in their 40s. Income cluster 3 (about 21% of respondents) displays patterns of persistently low or middle family income (“persistently low-middle income”), with family income starting mainly in the first or second quartiles and then staying mostly in the second quartile throughout. The remaining 20% of respondents, income cluster 4 (“persistently low income”), typically remained in the first family income quartile. Cluster 4 is perhaps the most homogenous, as most movements out of the lower-income quartile were short-lived and infrequent.

### Interrelation between work and income

Table 1 also presents the share of respondents with various joint work-and-family-income patterns. Row percentages, in parentheses, represent the share of respondents with a specific work pattern in each family income cluster. Over one-third of respondents with “mainly NW” work patterns (35%) had a family income pattern of “persistently low income,” and close to 20% had a family income pattern of “upward to high-income,” suggesting a somewhat bimodal distribution of people in this work cluster. In contrast, 36% and 29% of respondents with “mainly ST” work patterns had family income patterns of either “upward to high-income” or “upward to middle-income,” respectively. Moreover, about 30% of respondents with an “ST to mainly evenings/nights” work pattern had a family income pattern of “upward to middle-income,” with the remaining 70% equally distributed among the other three family income patterns. Finally, about one-third of respondents with the “ST to variable to ST” work pattern had a family income pattern of “upward to high-income,” with another 28% having an “upward to middle-income” pattern. Still, noticeable shares of respondents in “ST to variable to ST” had family income patterns of “persistently low-middle income” (22%) or “persistently low income” (17%).

Appendix Tables 1 and 2 (see Additional Files) present sociodemographic characteristics and outcome variables for the total sample and by the four clusters of work patterns (Appendix Table 1) and family income patterns (Appendix Table 2). The differences in sociodemographic characteristics between work and income patterns confirm the relatively less advantaged background associated with “mainly NW” and with “persistently low income” or “persistently low-middle income.”

### Associations between work-income patterns and health outcomes^1^

Tables 2, 3, and 4 present regression estimates of work and income patterns on health outcomes at age 50 after considering sociodemographic characteristics. All analyses use respondents in respective cluster 2 as the reference group (i.e., “mainly ST” and “upward to high-income”) because of their largest representation in the sample. We present three models for each regression analysis of the outcome variables: Model 1 only considers work and income trajectories (each controlling only for the other); Model 2 adds controls for sociodemographic characteristics by age 22; and Model 3 further adds controls for characteristics between ages 22 and 49. The changes in trajectory estimates across these three models allow us to gauge how sociodemographic characteristics might explain these associations by age 22 and between ages 22 and 49. This sequential approach is helpful given that work and income patterns might be shaped by characteristics before age 22 that might also influence health outcomes at age 50. In contrast, time-varying characteristics between ages 22 and 49 might be significant confounders for the associations between work and income trajectories and health outcomes at age 50.

Table 2 presents results for SF-12 physical functions at age 50. For Model 1, respondents in all work clusters and income clusters had significantly negative associations with physical functions at age 50, in comparison to the reference clusters (“mainly ST”/ “upward to high-income”). After considering sociodemographic characteristics before age 22 (Model 2), all work and income clusters remain statistically significant with somewhat smaller coefficient estimates, suggesting some negative associations are related to the correlations between sociodemographic characteristics and work and income, which is expected (selection effect). When considering characteristics between ages 22 and 49 (Model 3), all work and income clusters remain statistically significant, though coefficient estimates again become somewhat smaller. Specifically, Model 1 estimates indicate that having a “mainly NW” work pattern is associated with significantly lower physical function by three-tenths of a standard deviation compared to having a “mainly ST” work pattern, whereas having “persistently low income” between ages 22 and 49 is associated with significantly lower physical function by seven-tenths of a standard deviation compared to those in the “upward high-income” cluster. These effect sizes become smaller in Model 3 after considering sociodemographic characteristics before age 22 and between ages 22 and 49; having a “mainly NW” work pattern is related to significantly lower physical function by slightly over two-tenths of a standard deviation compared to having a “mainly ST” work pattern, whereas having “persistently low income” between ages 22 and 49 is related to significantly lower physical function by five-tenths of a standard deviation compared to those in the “upward to high-income” cluster. Note that the adjusted R-squared does not change much between Models 2 and 3, suggesting that adding characteristics between ages 22 and 49 does not explain much of the variation beyond what the sociodemographic characteristics before age 22 could explain in physical functions at age 50.

**Table 2:** Regression estimates of work schedules and income clusters on SF-12 physical functions at age 50, NLSY79 (*N* = 6,666)

	Model 1		Model 2		Model 3	
	b (sd)	sig.	b (sd)	sig.	b (sd)	sig.
Work schedule patterns (ref: Mainly ST)						
Mainly NW	-3.957 (0.501)	***	-3.330 (0.500)	***	-2.549 (0.568)	***
ST to evenings/nights	-1.492 (0.431)	***	-1.404 (0.427)	**	-1.295 (0.432)	**
ST to variable to ST	-1.468 (0.288)	***	-1.295 (0.286)	***	-1.136 (0.289)	***
Family income patterns (ref: Upward to high-income)						
Upward to middle-income	-2.242 (0.267)	***	-1.545 (0.278)	***	-1.410 (0.278)	***
Persistently low-middle income	-4.396 (0.320)	***	-3.292 (0.358)	***	-3.061 (0.367)	***
Persistently low income	-7.361 (0.400)	***	-5.743 (0.474)	***	-5.257 (0.487)	***
Age			-0.040 (0.054)		-0.034 (0.054)	
Being female			-0.959 (0.240)	***	-0.771 (0.283)	**
Race-ethnicity (ref: Non-Hispanic White)						
Non-Hispanic Black			0.566 (0.332)		0.507 (0.333)	
Hispanic			0.759 (0.399)		0.680 (0.399)	
Others			0.549 (0.948)		0.545 (0.957)	
Parental education at age 14 (ref: High school degree)						
Less than high school			-1.116 (0.315)	***	-1.142 (0.315)	***
Some college			-0.534 (0.388)		-0.535 (0.388)	
College or above			-0.227 (0.343)		-0.253 (0.342)	
Not living with bio-parents at age 14			0.302 (0.280)		0.331 (0.279)	
Residence at age 14 (ref: Urban)						
Suburban			0.137 (0.330)		0.135 (0.330)	
Rural			0.065 (0.565)		-0.001 (0.565)	
Immigrant status (ref: Third + generation)						
First generation			2.000 (0.548)	***	1.898 (0.549)	***
Second generation			0.482 (0.431)		0.428 (0.432)	
Having health conditions limiting work before age 22			-3.382 (0.480)	***	-3.392 (0.481)	***
Education by age 23 (ref: High school degree)						
Less than high school			-1.441 (0.399)	***	-1.378 (0.400)	***
Some college			0.060 (0.305)		-0.135 (0.314)	
College or above			1.566 (0.341)	***	1.122 (0.378)	**
Being a parent by age 22			-0.088 (0.371)		-0.166 (0.378)	
Marital status at age 22 (ref: Never married)						
Married			0.020 (0.318)		0.040 (0.319)	
Previously married			-0.864 (0.625)		-0.795 (0.622)	
Ever been poor by age 22			0.406 (0.274)		0.439 (0.275)	
Ever receiving welfare by age 22			-0.966 (0.397)	*	-0.965 (0.397)	*
Living in rural areas at age 22			-1.057 (0.334)	**	-1.031 (0.335)	**
Region of residence at age 22 (ref: South)						
Northeast			0.172 (0.343)		0.218 (0.344)	
Midwest			0.624 (0.316)	*	0.646 (0.317)	*
West			-0.168 (0.352)		-0.084 (0.353)	
# of marriages between ages 22–49					-0.186 (0.099)	
# of children between ages 22–49					0.113 (0.100)	
Primary occupation between ages 22–49 (ref: Service-related)						
Professional/managerial					0.599 (0.332)	
Sales-related					0.674 (0.714)	
Others					-0.203 (0.359)	
Mixed					-0.996 (0.588)	
Primary working hours between ages 22–49 (ref: Mainly full-time hours)						
Mainly part-time hours					-1.252 (0.413)	**
Mixed					-2.106 (1.001)	*
Intercept	53.168 (0.175)	***	53.949 (1.033)	***	53.825 (1.070)	***
Adjusted R-squared	0.100		0.127		0.129	

Note. NW: not working; ST: standard daytime hours. Numbers represent unstandardized coefficients with standard errors in parentheses. * *p* <.05, ** *p* <.01, *** *p* <.001

The estimates of work-income trajectories shown in Table 3 for SF-12 mental functions echo those for SF-12 physical functions, although weaker. For example, compared to having a “mainly ST” work pattern, having the “ST to evenings/nights” work pattern is not associated with significantly higher or lower mental function at age 50. Similarly, compared to the “upward to high-income” pattern, having the “upward to middle-income” pattern is not significantly related to higher or lower mental function at age 50. However, “upward to middle-income” is significantly associated with lower mental functions after controlling for sociodemographic characteristics before age 22 (Model 2), although the significance disappears when adding characteristics between ages 22 and 49 in Model 3. In terms of effect sizes, mental function at age 50 in Model 1 is two-tenths of a standard deviation lower for the “mainly NW” work pattern, dropping to about one-tenth of a standard deviation in Model 3, which considers characteristics before age 22 and between ages 22 and 49. In addition, mental function at age 50 in Model 1 is four-tenths of a standard deviation lower when being in the “persistently low income” cluster; the effect size is similar in Model 3, which additionally considers characteristics before age 22 and between ages 22 and 49. Similarly, the adjusted R-squared does not change much between Models 2 and 3, suggesting that adding characteristics between ages 22 and 49 does not explain much of the variation beyond the sociodemographic characteristics before age 22. However, we note that the adverse association between “upward to middle-income” and mental functions became nonsignificant with these additional controls.

**Table 3:** Regression estimates of work schedules and income clusters on SF-12 mental functions at age 50, NLSY79 (*N* = 6,666)

	Model 1		Model 2		Model 3	
	b (sd)	sig.	b (sd)	sig.	b (sd)	sig.
Work schedule patterns (ref: Mainly ST)						
Mainly NW	-2.351 (0.431)	***	-1.703 (0.434)	***	-1.037 (0.489)	*
ST to evenings/nights	0.167 (0.343)		0.088 (0.346)		0.188 (0.350)	
ST to variable to ST	-0.830 (0.257)	**	-0.742 (0.258)	**	-0.602 (0.261)	*
Family income patterns (ref: Upward to high-income)						
Upward to middle-income	-0.456 (0.238)		-0.529 (0.249)	*	-0.446 (0.253)	
Persistently low-middle income	-1.141 (0.290)	***	-1.224 (0.316)	***	-1.040 (0.328)	**
Persistently low income	-4.095 (0.359)	***	-4.030 (0.424)	***	-3.605 (0.431)	***
Age			-0.114 (0.047)	*	-0.109 (0.047)	*
Being female			-1.612 (0.220)	***	-1.394 (0.254)	***
Race-ethnicity (ref: Non-Hispanic White)						
Non-Hispanic Black			1.248 (0.291)	***	1.152 (0.291)	***
Hispanic			1.238 (0.347)	***	1.134 (0.347)	**
Others			-0.216 (1.148)		-0.216 (1.156)	
Parental education at age 14 (ref: High school degree)						
Less than high school			0.279 (0.273)		0.249 (0.273)	
Some college			-0.582 (0.359)		-0.578 (0.357)	
College or above			-0.802 (0.322)	*	-0.815 (0.323)	*
Not living with bio-parents at age 14			0.109 (0.248)		0.143 (0.247)	
Residence at age 14 (ref: Urban)						
Suburban			0.575 (0.277)	*	0.558 (0.276)	*
Rural			0.652 (0.474)		0.596 (0.473)	
Immigrant status (ref: Third + generation)						
First generation			-0.480 (0.535)		-0.551 (0.535)	
Second generation			0.282 (0.389)		0.282 (0.390)	
Having health conditions limiting work before age 22			-0.921 (0.408)	*	-0.913 (0.408)	*
Education by age 23 (ref: High school degree)						
Less than high school			-0.939 (0.350)	**	-0.918 (0.351)	**
Some college			0.184 (0.269)		0.045 (0.275)	
College or above			0.353 (0.333)		0.081 (0.351)	
Being a parent by age 22			-0.031 (0.323)		-0.136 (0.328)	
Marital status at age 22 (ref: Never married)						
Married			0.291 (0.273)		0.301 (0.274)	
Previously married			-1.454 (0.578)	*	-1.353 (0.578)	*
Ever been poor by age 22			-0.037 (0.243)		-0.009 (0.242)	
Ever receiving welfare by age 22			-0.729 (0.352)	*	-0.702 (0.352)	*
Living in rural areas at age 22			-0.207 (0.284)		-0.206 (0.285)	
Region of residence at age 22 (ref: South)						
Northeast			-0.604 (0.324)		-0.593 (0.325)	
Midwest			0.156 (0.284)		0.128 (0.285)	
West			0.178 (0.309)		0.206 (0.311)	
# of marriages between ages 22–49					-0.304 (0.088)	***
# of children between ages 22–49					0.145 (0.086)	
Primary occupation between ages 22–49 (ref: Service-related)						
Professional/managerial					0.399 (0.304)	
Sales-related					0.105 (0.618)	
Others					-0.058 (0.317)	
Mixed					0.406 (0.467)	
Primary working hours between ages 22–49 (ref: Mainly full-time hours)						
Mainly part-time hours					-1.323 (0.355)	***
Mixed					-0.794 (0.816)	
Intercept	54.530 (0.172)	***	57.096 (0.932)	***	57.061 (0.962)	***
Adjusted R-squared	0.043		0.063		0.067	

Note. NW: not working; ST: standard daytime hours. Numbers represent unstandardized coefficients with standard errors in parentheses. * *p* <.05, ** *p* <.01, *** *p* <.001

Table 4 presents the regression estimates of work–income trajectories on the likelihood of having at-risk depressive symptoms at age 50. Similar to results for SF-12 physical functions, all work and family income patterns have a significantly higher likelihood of having at-risk depressive symptoms compared to their respective reference groups. Adding characteristics before age 22 and between ages 22 and 49 makes the coefficient estimates smaller though remaining statistically significant.

**Table 4:** Regression estimates of work schedules and income clusters on CES-D depressive symptoms at age 50, NLSY79 (*N* = 6,672)

	Model 1		Model 2		Model 3	
	b (sd)	sig.	b (sd)	sig.	b (sd)	sig.
Work schedule patterns (ref: Mainly ST)						
Mainly NW	0.572 (0.105)	***	0.418 (0.108)	***	0.335 (0.124)	**
ST to evenings/nights	0.319 (0.113)	**	0.339 (0.116)	**	0.317 (0.117)	**
ST to variable to ST	0.321 (0.082)	***	0.283 (0.084)	***	0.256 (0.085)	**
Family income patterns (ref: Upward to high-income)						
Upward to middle-income	0.356 (0.100)	***	0.259 (0.105)	*	0.237 (0.107)	*
Persistently low-middle income	0.756 (0.100)	***	0.632 (0.113)	***	0.579 (0.118)	***
Persistently low income	1.299 (0.100)	***	1.146 (0.124)	***	1.054 (0.130)	***
Age			0.035 (0.016)	*	0.035 (0.017)	*
Being female			0.445 (0.076)	***	0.389 (0.090)	***
Race-ethnicity (ref: Non-Hispanic White)						
Non-Hispanic Black			-0.396 (0.097)	***	-0.361 (0.097)	***
Hispanic			-0.365 (0.119)	**	-0.341 (0.119)	**
Others			-0.223 (0.361)		-0.240 (0.364)	
Parental education at age 14 (ref: High school degree)						
Less than high school			-0.032 (0.087)		-0.020 (0.087)	
Some college			0.142 (0.117)		0.139 (0.117)	
College or above			0.104 (0.121)		0.103 (0.122)	
Not living with bio-parents at age 14			-0.001 (0.078)		-0.014 (0.079)	
Residence at age 14 (ref: Urban)						
Suburban			-0.106 (0.097)		-0.095 (0.098)	
Rural			-0.092 (0.168)		-0.075 (0.168)	
Immigrant status (ref: Third + generation)						
First generation			-0.189 (0.191)		-0.169 (0.191)	
Second generation			-0.083 (0.134)		-0.084 (0.134)	
Having health conditions limiting work before age 22			0.344 (0.105)	**	0.344 (0.105)	**
Education by age 23 (ref: High school degree)						
Less than high school			0.292 (0.094)	**	0.299 (0.094)	**
Some college			-0.143 (0.097)		-0.115 (0.099)	
College or above			-0.526 (0.150)	***	-0.468 (0.156)	**
Being a parent by age 22			0.143 (0.097)		0.175 (0.100)	
Marital status at age 22 (ref: Never married)						
Married			-0.051 (0.091)		-0.057 (0.092)	
Previously married			0.147 (0.143)		0.115 (0.143)	
Ever been poor by age 22			0.046 (0.083)		0.042 (0.083)	
Ever receiving welfare by age 22			0.005 (0.099)		-0.009 (0.100)	
Living in rural areas at age 22			0.097 (0.091)		0.102 (0.092)	
Region of residence at age 22 (ref: South)						
Northeast			0.093 (0.102)		0.103 (0.103)	
Midwest			-0.151 (0.095)		-0.129 (0.096)	
West			0.068 (0.103)		0.078 (0.104)	
# of marriages between ages 22–49					0.098 (0.026)	***
# of children between ages 22–49					-0.041 (0.029)	
Primary occupation between ages 22–49 (ref: Service-related)						
Professional/managerial					-0.104 (0.114)	
Sales-related					-0.069 (0.240)	
Others					-0.029 (0.100)	
Mixed					-0.242 (0.149)	
Primary working hours between ages 22–49 (ref: Mainly full-time hours)						
Mainly part-time hours					0.225 (0.101)	*
Mixed					0.198 (0.213)	
Intercept	-2.380 (0.079)	***	-3.029 (0.321)	***	-3.036 (0.330)	***
Pseudo R-squared	0.050		0.073		0.076	

Note. NW: not working; ST: standard daytime hours. Numbers represent unstandardized coefficients with standard errors in parentheses. * *p* <.05, ** *p* <.01, *** *p* <.001

### What might health look like with various work and family income patterns?

**Fig. 3:**
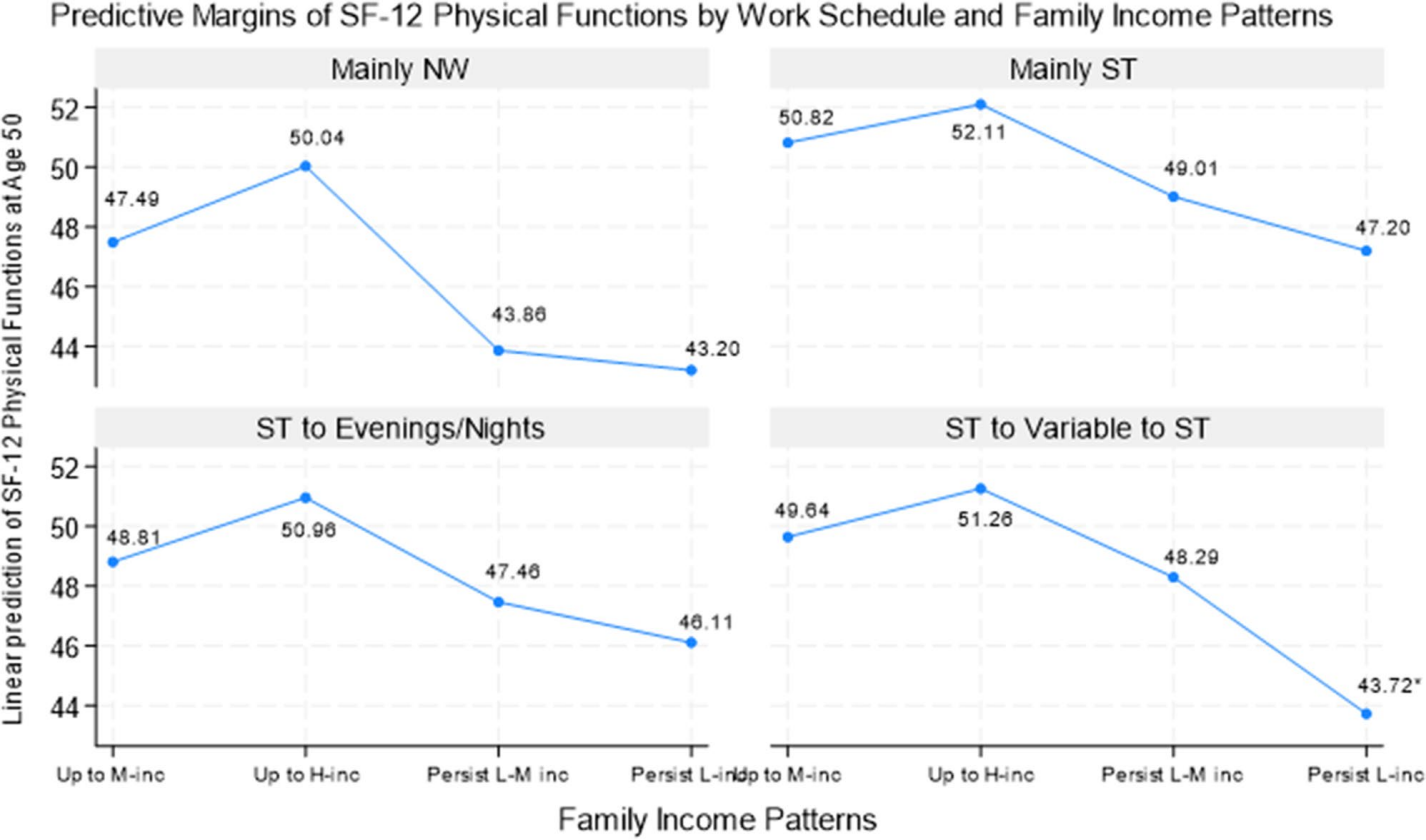
Predictive Margins of SF-12 Physical Functions at Age 50 By Work and Family Income Patterns, NLSY79. Note. NW: not working; ST: standard daytime hours; M-inc: middle-income; H-inc: high-income; L-M inc: low-middle income; L-inc: low-income. Predicted estimates are based on interaction analyses of work schedules by family income (results not shown, available upon request). Numbers with asterisks represent the predicted estimates that were statistically significantly different from the reference group at least at the 5% level

**Fig. 4:**
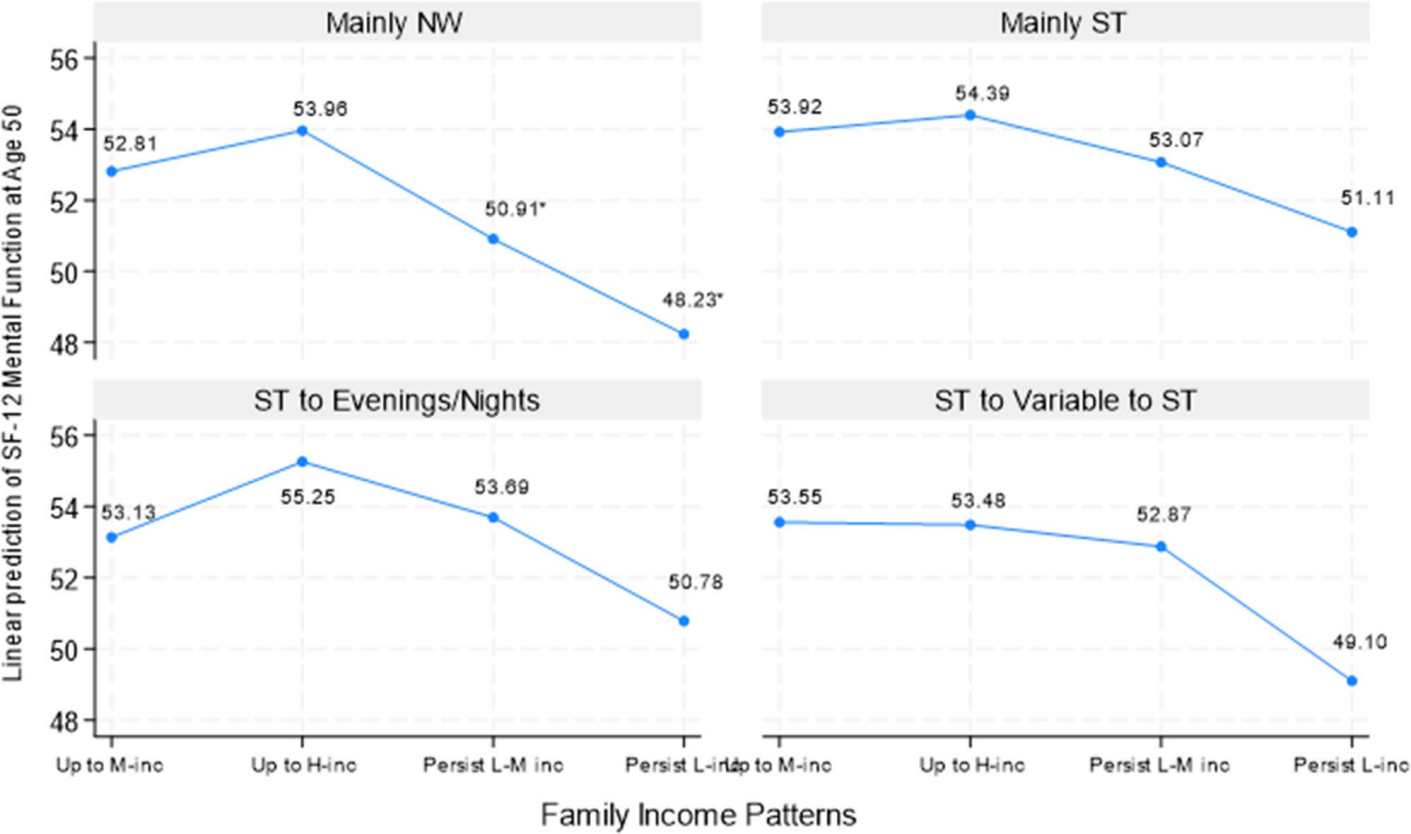
Predictive Margins of SF-12 Mental Functions at Age 50 By Work and Family Income Patterns, NLSY79. Note. NW: not working; ST: standard daytime hours; M-inc: middle-income; H-inc: high-income; L-M inc: low-middle income; L-inc: low-income. Predicted estimates are based on interaction analyses of work schedules by family income (results not shown, available upon request). Numbers with asterisks represent the predicted estimates that were statistically significantly different from the reference group at least at the 5% level

**Fig. 5:**
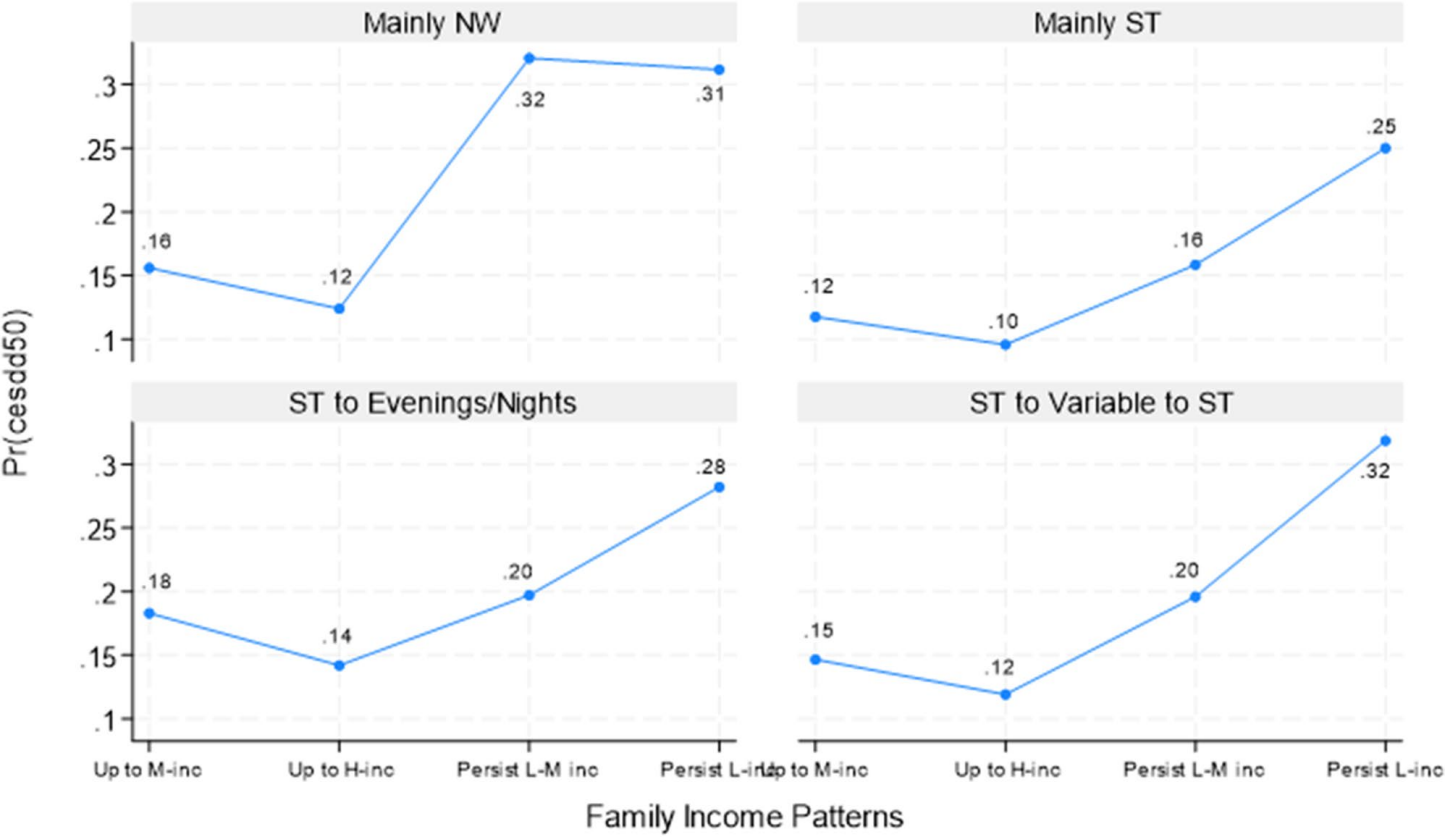
Predictive Margins of CES-D Depressive Symptoms at Age 50 By Work and Family Income Patterns, NLSY79. Note. NW: not working; ST: standard daytime hours; M-inc: middle-income; H-inc: high-income; L-M inc: low-middle income; L-inc: low-income. Predicted estimates are based on interaction analyses of work schedules by family income (results not shown, available upon request). Numbers with asterisks represent the predicted estimates that were statistically significantly different from the reference group at least at the 5% level

To understand the intersections of various work and family income patterns on health, we extended Models 2 and 3 with interactions of work and family income patterns. Figures 3, 4 and 5 display the predicted marginal effects of work and family patterns on SF-12 physical function (Fig. 3), SF-12 mental function (Fig. 4), and at-risk depressive symptoms (Fig. 5) based on these interaction analyses using Model 3 (results are almost the same whether using Models 2 or 3; results in tabulate form not shown, available upon request). Respondents had an average physical function (a score of approx. 49 to 50) when they had either the “upward to middle-income” or “upward to high-income” patterns. Indeed, those with “upward to high-income” had the highest physical function scores overall, regardless of work patterns. Physical function was relatively poorer for the “persistently low-middle income” or “persistently low income” groups. Specifically, respondents who were either “persistently low-middle income” or “persistently low income” and had “mainly NW” work patterns had the lowest physical functions out of all possible combinations of work and family income patterns (43.86 and 43.20, respectively). Those with the “persistently low income” pattern and the “ST to variable to ST” work trajectory also had one of the lowest average physical function scores (43.72). Conversely, those with an “upward to high-income” trajectory and either “mainly ST” (52.11) or “ST to variable to ST” work patterns (51.26) fared better. The difference between the lowest and highest physical function scores was 9 points, almost one standard deviation. In other words, as we move from left to right on income patterns, physical function scores tend to increase slightly, followed by substantial decreases. In contrast, the main adverse effects of “mainly NW” in Table 2 were primarily driven by having either the “persistently low-middle income” or “persistently low income” patterns. Similarly, the main effect of “ST to variable to ST” was primarily driven by the interaction with “persistently low income.”

Figure 4 presents the predicted margins of mental functions at age 50, with results largely following those in Fig. 3. Again, those who were “persistently low income” and had “mainly NW” or “ST to variable to ST” work patterns had the lowest mental function scores, 48.23 and 49.10, respectively. In contrast, those who had “upward to high-income” and “ST to evenings/nights” patterns had the highest mental function, 55.25, followed by those in the same income pattern but with the “mainly ST” work pattern (54.39). The difference between the lowest and the highest mental function scores was 7 points, or seven-tenths of a standard deviation. Similar to the results on physical function, the “persistently low-middle income” and “persistently low income” patterns in the “mainly NW” group were the primary drivers of the strong negative effect of “mainly NW” on mental function in Table 3.

Figure 5 displays the predicted likelihood of having at-risk depressive symptoms at age 50. Again, the results are similar to Figs. 3 and 4, although here, the higher the likelihood, the worse the outcome. Again, respondents with “persistently low-middle income” or “persistently low income” were the most likely to have at-risk depressive symptoms no matter their work patterns; the probabilities were, respectively,.32 and.31 for those with “mainly NW.” The corresponding values were.32 for those with “ST to variable to ST,”.28 for those with “ST to evenings/nights,” and.25 for those with “mainly ST” when they had “persistently low income.” The difference between the highest and the lowest probability (those with “upward to high income” and “mainly ST”) of having at-risk depressive symptoms was 0.22 (0.32–0.10). For depressive symptoms, the main effects of work and income were not moderated by each other statistically, with only one suggestive interaction between “mainly NW” and “persistently low-middle income” (*p* = 0.06; see Fig. 5, top-left panel).

### Do associations between work and income patterns vary by social position?

We next interacted work patterns with gender, race-ethnicity, and education, one at a time. We repeated the analyses for income patterns. Interaction regression estimates, presented in Appendix Tables 3, 4, 5, 6, 7 and 8 show the predicted marginal effects of work patterns (top panel) and income patterns (bottom panel) by gender, race-ethnicity, and education on the three health outcomes (see Additional Files).

**Fig. 6:**
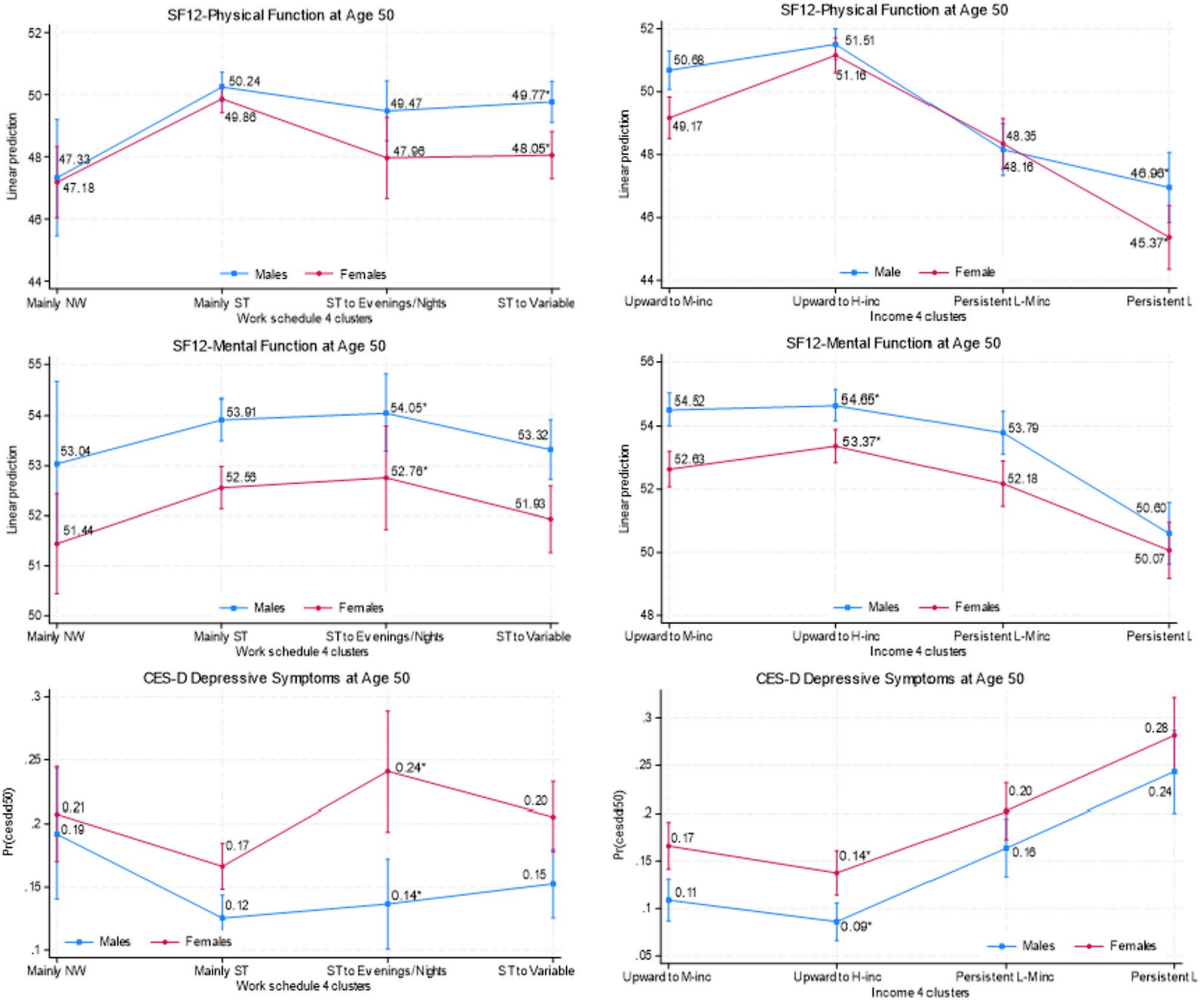
Predictive Margins of Work and Family Income by Gender with 95% Cis. *Note*. Numbers with asterisks indicate that the difference between those two numbers is statistically significant at least at the 5% level

In general, as shown in Fig. 6 and Appendix Tables 1 and 3, whereas females tended to have poorer health outcomes at age 50 than males (i.e., lower physical and mental function scores and a higher likelihood of depressive symptoms), these poorer health outcomes were more pronounced for physical function when females had a work pattern of “ST to variable to ST” (48.05 vs. 49.77 for males) or an income pattern of “persistently low-income” (45.37 vs. 46.96 for males), and for mental function and depressive symptoms when they had a work pattern of “ST to evenings/nights” (52.76 vs. 54.05 for males; 0.24 vs. 0.14 for males) or an income pattern of “upward to high-income” (53.37 vs. 54.65 for males; 0.14 vs. 0.09 for males). The omnibus tests (Appendix Table 3 in Additional Files) suggest interaction effects between income and gender on physical health.

**Fig. 7:**
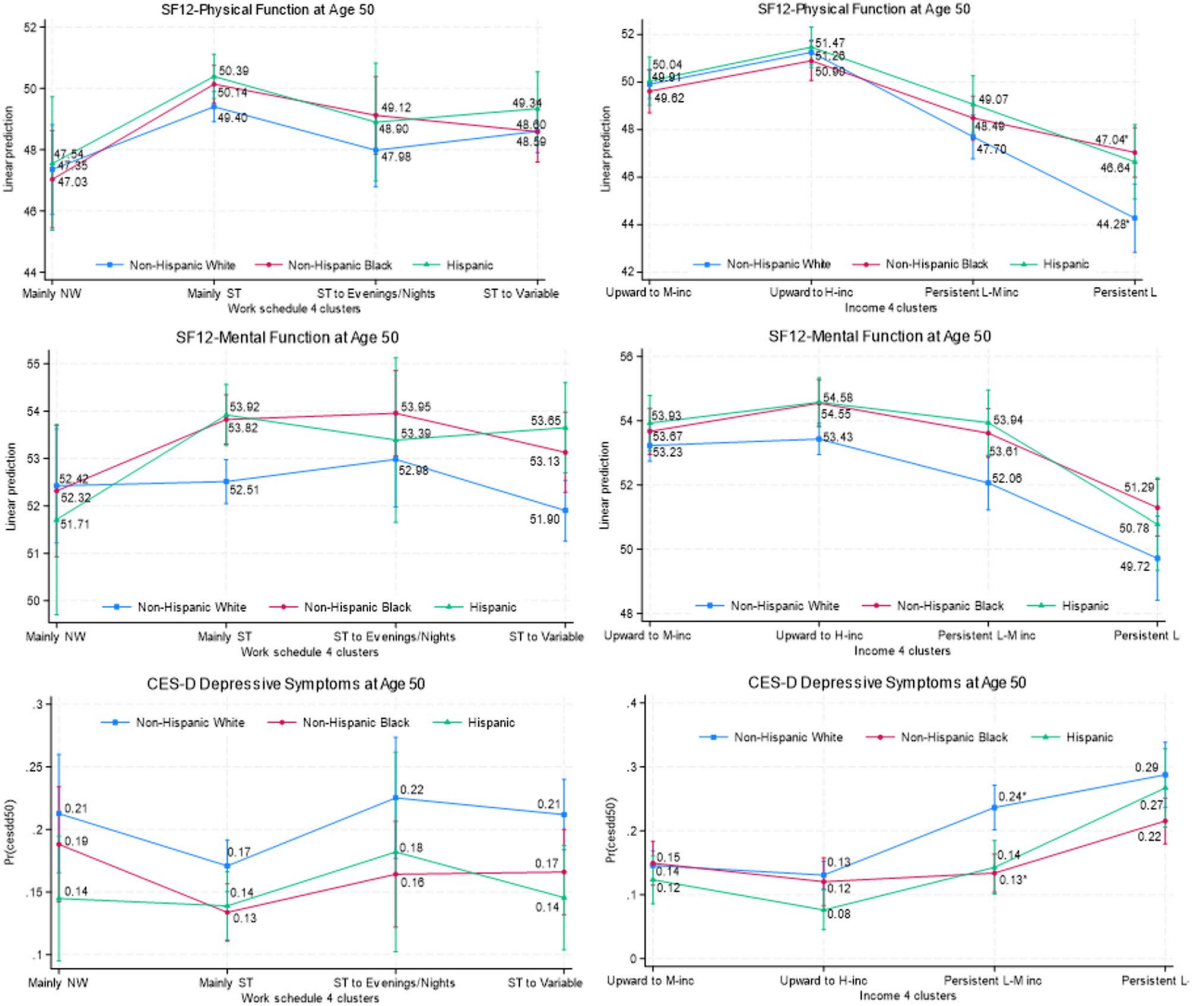
Predicted Margins of Work and Family Income By Race-Ethnicity with 95% Cis. *Note*. Numbers with asterisks indicate that the difference between those two numbers is statistically significant at least at the 5% level

Black respondents generally reported better health outcomes at age 50 than their White counterparts. Results were particularly pronounced for physical function when they had an income pattern of “persistently low income” (47.04 for Blacks vs. 44.28 for Whites) and for depressive symptoms when they had an income pattern of “persistently low-middle income” (0.13 for Blacks vs. 0.24 for Whites) (Fig. 7 and Appendix Tables 4 and 7). The omnibus tests suggest that the associations between race-ethnicity and depressive symptoms interact with income pathways.

**Fig. 8:**
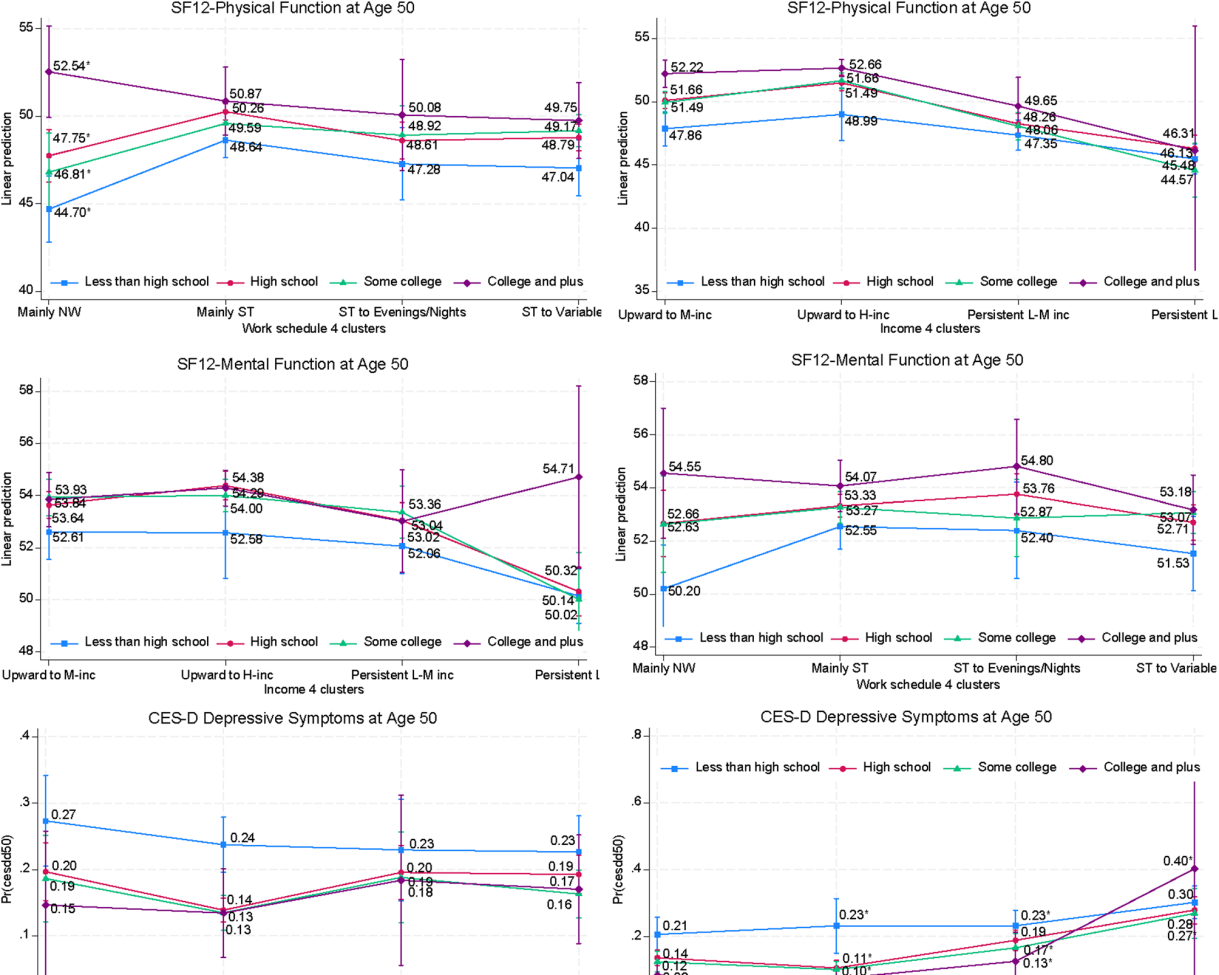
Predictive Margins of Work and Family Income by Education with 95% Cis. *Note*. Numbers with asterisks indicate that the difference between those two numbers is statistically significant at least at the 5% level

Individuals with less than a high school education reported the poorest health outcomes, regardless of their work or income patterns. This result was particularly pronounced for physical function (44.70) among those with a “mainly NW” work pattern and for depressive symptoms (0.23) among those with income patterns of “upward to high-income” or “persistently low-middle income” (Fig. 8 and Appendix Tables 5 and 8). While the results show that people with college or higher education had a very high probability of having depressive symptoms when they had a “persistently low income” pattern, this result was for a small group. Overall, omnibus tests identified significant interactions of education and work pathways on physical health.

## Discussion and conclusion

Work not only defines who we are but also shapes resources that determine general well-being, in turn, carrying significant implications for intergenerational economic stability and inequality. This paper conceptualizes work and family income as a joint “process” throughout our prime working ages to shed light on the interplay between work and resources (proxied by family income) in determining our long-term health. We also acknowledge the critical role of social position in shaping not only our work and family income trajectories but also the differing effects of work and family income on our long-term health. Through sequence analysis and a life course lens, we reveal the dynamic changes and transitions embedded in the diverse work and family income profiles experienced by the U.S. NLSY79 cohort over 25 years of their working lives, as well as how these diverse work and family income profiles might shape physical and mental health as we approach middle age (age 50).

Our sequence analysis results show relatively diverse work and family income patterns ranging from a stable work trajectory (e.g., “stable ST”) and upward income trajectories (e.g., “upward to middle-income” or “upward to high-income”) to a volatile work trajectory (e.g., “mainly NW” or “ST to variable to ST”) or disadvantaged family income trajectory (e.g., “persistently low income”). Indeed, the combination of “mainly NW” or “ST to variable to ST” with “persistently low-middle income” or “persistently low income” represents relatively poorer, if not the poorest, health outcomes for our NLSY79 cohorts. The adverse associations of health with volatile work and persistently low-income patterns produce effect sizes ranging from one-third to over half of a standard deviation.

Taken together, our results demonstrate a strong family income gradient in health that may be moderated by different work patterns. For example, having a “mainly NW” work trajectory would have had a score of 44.46 for physical function, 49.80 for mental function, and a 0.29 likelihood of having at-risk depressive symptoms. The corresponding figures would be 50.04, 53.96, and 0.12, respectively, should these individuals also have had an upward income mobility trajectory (e.g., upward to high-income). In contrast, these corresponding figures would be 43.20, 48.23, and 0.31 should these individuals also have had a persistently low-income trajectory. Similarly, having an “ST to variable to ST” work trajectory would have had a score of 48.95 for physical function, 52.68 for mental function, and a 0.18 likelihood of having at-risk depressive symptoms; the corresponding figures would be 51.26, 53.48, and 0.12 should these individuals have had an “upward to high-income” trajectory, but 43.72, 49.10, and a 0.32 should they have had a “persistently low income” trajectory — starkly contrasting predicted outcomes. In other words, family income is critical in cushioning the otherwise adverse effects on health of engaging in volatile work patterns, whereas limited or volatile income seems only to exacerbate the negative effects of these volatile work patterns on health.

Note that the literature suggests individuals with work trajectories other than primarily stable standard daytime hours are likely to have a relatively more disadvantaged long-term health risk profile [3, 6, 15]. Our sequence analysis plots (Figs. 1 and 2 and Appendix Figs. 1 and 2 in Additional Files) suggest that the NLSY79 respondents with a work pattern of “ST to evenings/nights” or “ST to variable to ST” were quite heterogeneous, likely comprising at least two diverse groups: (1) those who started with standard daytime hours during their 20s and (2) those who started with schedules other than standard daytime hours (e.g., evenings, nights, variable hours, or not working). The former possibly consisted of people with relatively advantaged characteristics while the latter had relatively disadvantaged characteristics (as the bimodal distribution in family income trajectories illustrated in Table 1). The effects of having the “ST to evenings/nights” or “ST to variable and to ST” work pattern on health were likely to be masked by the partly positive effects associated with the group with relatively advantaged backgrounds and the negative effects associated with those with relatively disadvantaged backgrounds. Still, we note that the adverse effects on health associated with the “ST to variable to ST,” a relatively volatile work pattern, were strong enough to appear in our analysis, suggesting stronger negative effects for an important subgroup (those who did not start on a standard daytime schedule in their 20s). These adverse work schedule effects were particularly strong for female respondents.

As expected, those with low education (e.g., no high school degree) had poorer health than their respective counterparts. Our results strongly suggest an educational gradient in health, with higher education associated with better health. In addition, females generally reported poorer health than males. Females with an “ST to variable to ST” work pattern or “persistently low income” trajectory reported significantly poorer physical function than males, and females with “ST to evenings/nights” or “upward to high-income” trajectories reported significantly poorer mental function and depressive symptoms than males. Researchers have examined biological (e.g., genetics) and sociological (e.g., family responsibilities, work) factors that may explain relatively poor health among women with unpredictable or insecure employment. One study found that juggling family responsibilities (e.g., childrearing) and work demands (e.g., non-daytime hours with low pay) were the most important factors [72]. For example, despite having similar work hours, women generally spend more time doing household work than men; these differences in household chores may influence time for sleep or self-care, impacting physical and emotional health [40]. Our results support this research. Indeed, females with nonstandard work schedules were more likely than males to report sleep disorders [72] and mental health symptoms [73]. Recent studies have suggested that gender inequality in the division of labor at home (household maintenance, childrearing) and in the workplace (e.g., unfavorable hours or tasks) is shaped by structural sexism [40]. These gender inequalities might then influence women’s physical and mental health, increasing women’s exposure to stress, further compounded by constrained access to material resources [40, 43]. In contrast, Black respondents, in general, reported similar physical health as Whites but better mental health, echoing the literature on the “Black-White mental health paradox” [74, 75]. The Black-White difference was particularly striking and statistically significant on physical function for the “persistently low income” trajectory and on depressive symptoms for the “persistently low-middle income” trajectory.

### Limitations

Our observational study has several limitations. First, the NLSY79 collected work schedule information annually until 1994 and biennially thereafter. For some, work schedules may have changed month to month, let al.one during the two-year windows, limiting our ability to depict more precise work patterns over time. Thus, our results likely underestimate volatility or work status shifts and the true association between work patterns and health. However, the longitudinal approach has the advantage of reducing measurement noise. Specifically, longitudinal data captures long-running patterns more accurately than cross-sectional data, such as individuals who repeatedly reported nonstandard work schedules over the years versus those who only worked such a schedule a few times in 30 years. Second, due to the data at hand, we could not distinguish whether individuals voluntarily chose specific work schedules, as the literature has clearly shown that voluntary choices tend to accrue positive outcomes while involuntary requirements accrue negative outcomes [45]. This limitation might be particularly relevant for NLSY79 respondents who chose to work variable/irregular hours. We found that respondents in the “ST to variable to ST” group had a bimodal income distribution echoed in the regression results on health outcomes: people with relatively advantaged characteristics generally reported better health. Nonetheless, the sociodemographic profiles associated with variable work patterns provide insight into how variable hours may induce opportunities versus constraints for different sociodemographic groups. Third, people may change their work status or schedules due to worsened health associated with work or working nonstandard hours. If so, we might underestimate the associations between work patterns and health. Similarly, while we control for health limitations before the start of the work trajectories to address potential reverse causality, individuals could develop poor health between data collection periods and subsequently change to a nonstandard work schedule in the following survey year. In such a case, poor health outcomes observed at age 50 might be erroneously attributed to the nonstandard work schedule. Our current analytic approach cannot capture this bidirectional relationship between health and work, but it is an area that would benefit from future investigation. Fourth, we focused on family income instead of individual income to fully capture the economic resources available to individuals, with the added benefit of allowing our analysis to be comparable with a large set of scholarship examining health using family income. Nonetheless, we conducted supplementary analyses using individual wages and interacting family income clusters with individual wage clusters (not shown, available upon request). The supplementary findings indicate that the significant negative associations of family income with health reported in this analysis were driven by and stronger for individuals with “persistently low wages” or “low-middle wages.” Respondents with an “upward to high-income” family income trajectory fared well health-wise even if they were not working. These supplementary analyses suggest a valuable avenue for future investigation. Fifth, we acknowledge that even with the large number of respondents to the NLSY79, we were not able to distinguish the Asian racial group from other non-White, non-Black, and non-Hispanic groups due to extremely small sample sizes in this “other” racial-ethnic group. This limitation warrants determined efforts to collect sufficient data for groups with small representation. Sixth, despite our analysis properly documenting sequential changes in work and income trajectories, our analyses at best represent associations, not causation. While an experimental design study is not feasible in this context, future analyses could enhance the sequence analysis with synthetic counterfactuals [76] to get one step closer to causation. Moreover, we took a conservative approach to forming the typology of work and income trajectories. While this approach ensured replicability, it sacrificed nuance, as subtle differences between trajectories may be important to outcomes. Future work could use different sequence analytic techniques that sacrifice cluster quality for predictive performance (cf [77]). Despite these limitations, the NLSY79 presents a unique opportunity, with its work schedule information for a nationally representative sample in the U.S. over three decades, to estimate how our lifetime work and income trajectories weigh on our long-term health.

We conclude our study by discussing the implications of how work and family income as SODH factors help shape intergenerational poverty and inequality. A recent review of empirical evidence found that low wages and family income perpetuate the cycle of economic disadvantage, in part by leaving families unable to provide their children with adequate nutrition and housing, access to medical care, and enrichment and learning activities, along with factors that might promote intergenerational economic stability [78]. Studies have shown that work with stable hours, decent pay, and job-associated benefits is essential for health and well-being [6], carrying significant implications for the well-being of the next generations through work-induced material and nonmaterial resources (e.g., better mental health). Indeed, studies examining policy changes over the past 25 years have provided strong evidence that income support policies (e.g., expanded Earned Income Tax Credit), when combined with quality employment, may allow families, particularly those in disadvantaged social positions, to produce consistent positive effects on child development, thereby reducing intergenerational poverty and inequality [78].

## Electronic supplementary material

Below is the link to the electronic supplementary material.

Supplementary Material 1: Appendix A. Empirical Strategies. Appendix B. Clustering Methodology. Appendix Fig. 1. Sequence Cluster Solutions on Work Between Ages 22–49, NLSY79 = 7,871. Appendix Fig. 2. Sequence Cluster Solutions on Family Income Between Ages 22–49, NLSY79 = 7,871. Appendix Table 1. Descriptive Statistics of Analyzed Variables by Work Patterns, NLSY79 (*N* = 6,672). Appendix Table 2. Descriptive Statistics of Analyzed Variables by Family Income Patterns, NLSY79 (*N* = 6,672). Appendix Table 3. Regression Estimates of Work and Income Patterns Interacted with Gender on Health Outcomes at Age 50 (NLSY79). Appendix Table 4. Regression Estimates of Work and Income Patterns Interacted with Race-Ethnicity on Health Outcomes at Age 50 (NLSY79). Appendix Table 5. Regression Estimates of Work and Income Patterns Interacted with Education on Health Outcomes at Age 50 (NLSY79). Appendix Table 6. Predictive Margins of Work and Income Patterns by Gender on Health Outcomes at Age 50. Appendix Table 7. Predictive Margins of Work and Income Patterns by Race-Ethnicity on Health Outcomes at Age 50. Appendix Table 8. Predictive Margins of Work and Income Patterns by Education on Health Outcomes at Age 50.

## Data Availability

No datasets were generated or analysed during the current study.

## Footnotes

1 empirically, family income trajectories are associated with health outcomes as they reflect available resources over time, and health effects are cumulative. In supplemental analyses (not shown, available upon request), we constructed personal wage trajectories over the same 22–49 age period to determine whether personal wages are associated with health beyond what work and family income would predict, and derived five categories using sequence analysis. We then added this person-level wage information in our models and found that the original conclusions hold, with the caveat that individuals experiencing low or limited wages tend to have even worse health outcomes than those with similar family income resources. Thus, the “protective” effect of high family income on later health May be attenuated for individuals who experience particularly low wages or extended periods of not working
