# Supplementary material for: Health as a rich people’s game through the lens of work and income
Source: BMC Public Health. 2025 May 31;25:2013. doi: 10.1186/s12889-025-23320-z (PMC12125729; doi:10.1186/s12889-025-23320-z)

**Appendix A on Empirical Strategy**

We first addressed the missing values associated with work and family income. Two primary sources of missing values for these two variables were (1) non-survey years (biennial data collection after 1994) and (2) nonresponse (either did not respond to the question or did not take part in the survey during that particular year). Our approach intends to address the first source of missing values and the nonresponse resulting from the respondents not providing information on the variable despite participating in that survey year. First, due to the survey design, all respondents were structurally missing every other year for about half of the survey years of interest. The ages at which they were missing varied in a non-overlapping manner. For identifying similar patterns in life course trajectories, structurally missing values induce slight timing differences across age cohorts that are expected to be inconsequential. For example, if one worked standard hours every survey year (e.g., ages 22, 24, 26), we expected this work pattern to be held during non-surveyed years (e.g., ages 23, 25, 27). Since the survey design induced the differences in timing, we imputed values between years to minimize individual differences. Data-driven methods exist for multiply-imputing state sequences [60], and we based our single imputation on that same stochastic algorithm but adjusted it to be more deterministic based on our substantive knowledge of labor market behavior. Regarding work and income, we note that observations changed slowly over time. Thus, the missing work schedule and family income observations were imputed using data from one and two years before and after the missing year with weights of 1/6, 1/3, 1/3, and 1/6, respectively. Work information is categorical, so we drew the modal from among available nearby observations. Ties led to random draws. This scheme resembles categorical data smoothing [61], which borrows strength from nearby cells in multiway tables. We followed a similar imputation procedure for family income but imputed family income quartiles instead of the raw income values, establishing a reasonably smooth progression in income while still allowing for instability (upward and downward mobility). Note that we converted income to quartiles across all people and time, so in theory, everyone could start in Q1 and end up in Q4 as they aged from 22 to 49 years old, but this does not happen. About 45% of family income and 32% of the work information were imputed.

Next, we conducted separate sequence analyses [62-64] for work and family income. Sequence analysis identifies predominant types of work and income patterns by establishing clusters. Key ingredients to a sequence analysis include distance measures for comparing pairs of trajectories (emphasizing the most important features), the clustering method, and the choice of the number of clusters. For pairwise distance, we chose the longest common subsequence (LCS) distance [64,65]. This distance measure tends to produce clusters that closely resemble each other in content, timing, and sequencing, which are all important in our study. LCS similarity is the length of the common subsequence formed by deleting mismatched states between the pairs. We used a well-established “divisive” method, the partitioning around medoids (PAM) [66] algorithm, for clustering. The ideal number of clusters was based on the goodness of fit measure known as the average silhouette width [67]. We compared these results to clustering using data-driven distances such as TRATE [65,68]. We also examined cluster solutions using other algorithms (e.g., Ward’s distance hierarchical clustering and partitioning method AGNES) as a robustness check. When choosing the number of clusters, we compared a variety of goodness of fit indices [69], including the point biserial correlation (PBC), Hubert’s gamma (HG), the average silhouette width (ASW), and Hubert’s C (HC) (ASW applies to PAM, while hierarchical clustering algorithms were used for the others). As a final robustness check, we used Hennig’s *clusterboot* routine, which uses bootstrap samples to assess the uncertainty in the clustering [70].

As a sensitivity check of our imputing scheme on the cluster solutions, we examined the pattern in the timing of missing observations cross-sectionally, by cluster, to look for differences that could drive the findings. Our examination (not shown, available upon request) indicates that the work variable showed no unusual imputation patterns. In contrast, the lowest income quartile was more likely to be imputed at younger ages in the persistent low-middle and persistent low-income clusters, indicating weak attachment to the labor force.

**Appendix B on Clustering Methodology**

Fit statistics (PBC, HG, ASW, HC) suggest four or five clusters, with ASW clearly favoring four. Hennig’s *clusterboot* routine, which examines the stability of a range of cluster solutions, strongly favors four clusters, with reasonably high Jaccard Indices of 0.90, 0.94, 0.64, 0.84 for work and 0.81, 0.92, 0.80, 0.94 for family income. Rand indices comparing the LCS distance, Wards method to TRATE, Agnes method, and our PAM-based solution are 0.77, 0.82, 0.78, respectively, for work, and 0.83, 0.88, 0.83, respectively, for family income clustering. We prefer the LCS distance PAM clustering method for its robustness [70].

Appendix Table 1. Descriptive Statistics of Analyzed Variables by Work Patterns, NLSY79 (N = 6,672)

|  |  | Work | Patterns |  |  |  |
| --- | --- | --- | --- | --- | --- | --- |
|  | Total | Mainly NW | Mainly ST | ST to Evenings/Nights | ST to Variable to ST | Sig. |
| Age | 17.60 (2.25) | 17.70 (2.25) | 17.60 (2.26) | 17.47 (2.19) | 17.59 (2.25) | ns |
| Female (%) | 51.98 | 72.40 | 49.71 | 45.05 | 49.22 | *** |
| Race-Ethnicity (%) |  |  |  |  |  | *** |
| Non-Hispanic White | 49.71 | 36.15 | 51.98 | 42.14 | 54.75 |  |
| Non-Hispanic Black | 29.84 | 40.73 | 25.98 | 43.33 | 27.48 |  |
| Hispanic | 19.30 | 22.10 | 20.78 | 13.74 | 16.69 |  |
| Others | 1.14 | 1.02 | 1.26 | 0.79 | 1.07 |  |
| Background at age 14 |  |  |  |  |  |  |
| Parental education (%) |  |  |  |  |  | *** |
| Less than HS | 34.13 | 47.03 | 32.49 | 40.44 | 28.90 |  |
| High school (HS) | 38.98 | 33.59 | 39.66 | 39.20 | 40.06 |  |
| Some college | 11.75 | 9.14 | 12.20 | 11.14 | 12.27 |  |
| College+ | 15.14 | 10.24 | 15.65 | 9.22 | 18.78 |  |
| Not living with bio parents (%) | 31.95 | 42.36 | 28.48 | 37.38 | 32.21 | *** |
| Living location (%) |  |  |  |  |  | *** |
| Urban | 79.40 | 83.98 | 78.29 | 81.01 | 78.88 |  |
| Suburban | 15.82 | 13.16 | 16.94 | 14.61 | 15.15 |  |
| Rural | 4.78 | 2.86 | 4.77 | 4.38 | 5.98 |  |
| Immigrant status (%) |  |  |  |  |  | *** |
| First-generation | 5.49 | 6.62 | 6.37 | 4.10 | 3.44 |  |
| Second generation | 9.01 | 10.49 | 8.90 | 7.79 | 8.96 |  |
| Third+ generation | 85.50 | 82.89 | 84.73 | 88.11 | 87.60 |  |
| Having health conditions limiting work before age 22 (%) | 9.72 | 16.70 | 8.01 | 9.64 | 9.98 | *** |
| Education by age 23 |  |  |  |  |  | *** |
| Less than HS | 20.64 | 40.55 | 17.84 | 19.26 | 17.12 |  |
| High school | 42.67 | 37.79 | 41.69 | 54.98 | 42.49 |  |
| Some college | 23.13 | 15.32 | 23.54 | 19.92 | 27.63 |  |
| College+ | 13.56 | 6.33 | 16.93 | 5.84 | 12.76 |  |
| Being a parent at age 22 (%) | 29.95 | 45.01 | 26.38 | 33.29 | 28.82 | *** |
| Marital status at age 22 (%) |  |  |  |  |  | * |
| Married | 28.45 | 28.31 | 28.46 | 27.34 | 28.93 |  |
| Previously married | 5.45 | 7.64 | 4.66 | 6.21 | 5.80 |  |
| Never married | 66.10 | 64.05 | 66.88 | 66.45 | 65.27 |  |
| Ever been poor by age 22 (%) | 44.05 | 62.32 | 39.92 | 49.01 | 41.87 | *** |
| Ever receiving welfare assistance by age 22 (%) | 22.20 | 43.28 | 16.77 | 26.55 | 21.74 | *** |
| Living in rural area at age 22 (%) | 18.46 | 16.09 | 18.15 | 22.06 | 18.95 | * |
| Region of residence at age 22 (%) |  |  |  |  |  | *** |
| Northeast | 18.14 | 21.38 | 18.18 | 19.02 | 16.00 |  |
| Midwest | 23.63 | 21.69 | 22.60 | 28.93 | 24.85 |  |
| South | 38.47 | 36.15 | 39.47 | 38.97 | 37.20 |  |
| West | 19.76 | 20.77 | 19.75 | 13.08 | 21.95 |  |
| Between ages 22-49 |  |  |  |  |  |  |
| # of marriages | 1.25 (1.28) | 1.06 (1.29) | 1.24 (1.24) | 1.32 (1.35) | 1.36 (1.31) | *** |
| # of children | 1.97 (1.37) | 2.41 (1.64) | 1.88 (1.27) | 1.96 (1.49) | 1.94 (1.35) | *** |
| Primary weekly working hours (%) |  |  |  |  |  |  |
| Full-time (>=35) | 75.07 | 11.61 | 88.03 | 80.58 | 76.60 | *** |
| Part-time (>0 & <35) | 22.74 | 84.62 | 10.26 | 17.44 | 20.88 | *** |
| Mixed | 2.19 | 3.77 | 1.71 | 1.98 | 2.52 | *** |
| Primary occupation (%) |  |  |  |  |  | *** |
| Prof./Managerial | 24.14 | 10.42 | 28.27 | 10.57 | 26.89 |  |
| Sales related | 2.45 | 2.96 | 2.46 | 0.40 | 3.01 |  |
| Service related | 36.29 | 52.30 | 31.39 | 47.69 | 35.05 |  |
| Others | 31.10 | 22.70 | 33.33 | 37.12 | 27.64 |  |
| Mixed | 6.02 | 11.62 | 4.54 | 4.23 | 7.41 |  |
| Outcomes at age 50 |  |  |  |  |  |  |
| SF12-physical function | 49.13 (10.23) | 44.08 (12.63) | 50.57 (8.93) | 48.01 (10.60) | 48.88 (10.61) | *** |
| SF12-mental function | 52.88 (8.81) | 49.62 (10.93) | 53.62 (8.08) | 53.16 (8.30) | 52.75 (9.02) | *** |
| CES-D >=8 (%) | 17.25 | 29.96 | 13.51 | 20.70 | 17.83 | *** |

*Note*. NW: not working; ST: standard daytime hours; ns=not statistically significant. Bivariate differences in sociodemographic characteristics between work schedule cluster patterns were conducted by either chi-square for categorical variables or t-test/ANOVA for continuous variables.

* *p* < .05, ** *p* < .01, *** *p* < .001.

Appendix Table 2. Descriptive Statistics of Analyzed Variables by Family Income Patterns, NLSY79 (N = 6,672)

|  |  | Family | Income | Patterns |  |  |
| --- | --- | --- | --- | --- | --- | --- |
|  | Total | Upward to Middle-Income | Upward to High-Income | Persistently Low-Middle Income | Persistently Low-Income | Sig. |
| Age | 17.60 (2.25) | 17.62 (2.27) | 17.59 (2.26) | 17.63 (2.23) | 17.55 (2.23) | ns |
| Female (%) | 51.98 | 50.16 | 50.48 | 50.30 | 58.41 | *** |
| Race-Ethnicity (%) |  |  |  |  |  | *** |
| Non-Hispanic White | 49.71 | 57.03 | 66.68 | 38.83 | 25.03 |  |
| Non-Hispanic Black | 29.84 | 22.90 | 16.12 | 37.38 | 52.49 |  |
| Hispanic | 19.30 | 19.04 | 15.75 | 22.83 | 21.48 |  |
| Others | 1.14 | 1.03 | 1.45 | 0.97 | 1.00 |  |
| Background at age 14 |  |  |  |  |  |  |
| Parental education (%) |  |  |  |  |  | *** |
| Less than HS | 34.13 | 32.03 | 16.28 | 45.11 | 54.86 |  |
| High school (HS) | 38.98 | 42.49 | 39.93 | 38.21 | 33.31 |  |
| Some college | 11.75 | 12.86 | 15.34 | 9.03 | 7.14 |  |
| College+ | 15.14 | 12.62 | 28.44 | 7.65 | 4.69 |  |
| Not living with bio parents (%) | 31.95 | 29.34 | 20.59 | 37.38 | 47.38 | *** |
| Living location (%) |  |  |  |  |  | *** |
| Urban | 79.40 | 77.46 | 81.26 | 76.92 | 81.66 |  |
| Suburban | 15.82 | 17.44 | 13.89 | 18.17 | 14.21 |  |
| Rural | 4.78 | 5.09 | 4.85 | 4.91 | 4.13 |  |
| Immigrant status (%) |  |  |  |  |  | ns |
| First-generation | 5.49 | 5.50 | 5.20 | 6.28 | 5.11 |  |
| Second generation | 9.01 | 8.84 | 8.42 | 9.54 | 9.59 |  |
| Third+ generation | 85.50 | 85.66 | 86.38 | 84.18 | 85.31 |  |
| Having health conditions limiting work before age 22 (%) | 9.72 | 8.74 | 6.85 | 9.84 | 15.32 | *** |
| Education by age 23 |  |  |  |  |  | *** |
| Less than HS | 20.64 | 14.74 | 5.29 | 26.94 | 45.75 |  |
| High school | 42.67 | 49.39 | 32.41 | 48.55 | 43.56 |  |
| Some college | 23.13 | 25.42 | 31.11 | 20.82 | 10.12 |  |
| College+ | 13.56 | 10.45 | 31.19 | 3.69 | 0.56 |  |
| Being a parent at age 22 (%) | 29.95 | 30.04 | 16.56 | 36.23 | 44.02 | *** |
| Marital status at age 22 (%) |  |  |  |  |  | *** |
| Married | 28.45 | 35.17 | 26.99 | 28.93 | 21.30 |  |
| Previously married | 5.45 | 5.08 | 3.42 | 6.88 | 7.60 |  |
| Never married | 66.10 | 59.76 | 69.58 | 64.19 | 71.11 |  |
| Ever been poor by age 22 (%) | 44.05 | 36.72 | 27.07 | 52.42 | 71.36 | *** |
| Ever receiving welfare assistance by age 22 (%) | 22.20 | 16.22 | 6.93 | 27.11 | 48.63 | *** |
| Living in rural area at age 22 (%) | 18.46 | 20.03 | 14.42 | 20.83 | 20.17 | *** |
| Region of residence at age 22 (%) |  |  |  |  |  | *** |
| Northeast | 18.14 | 16.41 | 22.52 | 16.97 | 14.88 |  |
| Midwest | 23.63 | 25.76 | 25.34 | 20.23 | 21.67 |  |
| South | 38.47 | 37.61 | 31.39 | 42.51 | 46.39 |  |
| West | 19.76 | 20.22 | 20.75 | 20.29 | 17.06 |  |
| Between ages 22-49 |  |  |  |  |  |  |
| # of marriages | 1.25 (1.28) | 1.30 (1.31) | 1.19 (1.10) | 1.39 (1.40) | 1.14 (1.34) | *** |
| # of children | 1.97 (1.37) | 1.98 (1.32) | 2.02 (1.20) | 1.87 (1.43) | 1.98 (1.62) | ** |
| Primary weekly working hours (%) |  |  |  |  |  |  |
| Full-time (>=35) | 75.07 | 84.11 | 82.76 | 82.73 | 43.34 | *** |
| Part-time (>0 & <35) | 22.74 | 13.96 | 15.63 | 15.10 | 53.24 | *** |
| Mixed | 2.19 | 1.93 | 1.61 | 2.17 | 3.42 | *** |
| Primary occupation (%) |  |  |  |  |  | *** |
| Prof./Managerial | 24.14 | 22.70 | 47.80 | 10.44 | 2.84 |  |
| Sales related | 2.45 | 1.84 | 4.60 | 1.09 | 1.29 |  |
| Service related | 36.29 | 35.56 | 25.21 | 41.69 | 49.26 |  |
| Others | 31.10 | 33.54 | 17.47 | 40.84 | 39.20 |  |
| Mixed | 6.02 | 6.36 | 4.92 | 5.95 | 7.41 |  |
| Outcomes at age 50 |  |  |  |  |  |  |
| SF12-physical function | 49.13 (10.23) | 50.17 (9.39) | 52.35 (7.49) | 47. 99 (10.51) | 43.78 (12.30) | *** |
| SF12-mental function | 52.88 (8.81) | 53.77 (7.71) | 54.15 (7.46) | 53.04 (9.14) | 49.49 (10.88) | *** |
| CES-D >=8 (%) | 17.25 | 13.57 | 9.86 | 18.83 | 32.42 | *** |

*Note*. NW: not working; ST: standard daytime hours; ns=not statistically significant. Bivariate differences in sociodemographic characteristics between work schedule cluster patterns were conducted by either chi-square for categorical variables or t-test/ANOVA for continuous variables.

* *p* < .05, ** *p* < .01, *** *p* < .001.

Appendix Table 3. Regression Estimates of Work and Income Patterns Interacted with Gender on Health Outcomes at Age 50 (NLSY79)

|  | SF-12 Physical Function |  | SF-12 Mental Function |  | CES-D Depressive Symptoms |  |
| --- | --- | --- | --- | --- | --- | --- |
|  | b (sd) | sig. | b (sd) | sig. | b (sd) | sig. |
| Work schedule Patterns (ref: Mainly ST) |  |  |  |  |  |  |
| Mainly NW | -2.917 (1.015) | ** | -0.875 (0.877) |  | 0.540 (0.208) | ** |
| ST to Evenings/Nights | -0.772 (0.525) |  | 0.136 (0.428) |  | 0.104 (0.180) |  |
| ST to Variable to ST | -0.479 (0.372) |  | -0.592 (0.337) |  | 0.243 (0.133) |  |
| Mainly NW x Female | 0.236 (1.130) |  | -0.239 (.994) |  | -0.254 (0.234) |  |
| ST to Evenings/Nights x Female | -1.128 (0.872) |  | 0.064 (0.701) |  | 0.386 (0.236) |  |
| ST to Variable to ST x Female | -1.333 (0.572) | * | -0.031 (0.514) |  | 0.029 (0.171) |  |
| Family income patterns (ref: upward to high-income) |  |  |  |  |  |  |
| Upward to middle-income | -0.825 (0.354) | * | -0.133 (0.315) |  | 0.265 (0.169) |  |
| Persistently low-middle income | -3.350 (0.477) | *** | -0.860 (0.411) | * | 0.750 (0.175) | *** |
| Persistently low-income | -4.552 (0.653) | *** | -4.050 (0.578) | *** | 1.265 (0.188) | *** |
| Upward to middle-income x Female | **-1.160 (0.532)** | * | -0.602 (0.476) |  | -0.038 (0.209) |  |
| Persistently low-middle income x Female | **0.541 (0.646)** |  | -0.333 (0.582) |  | -0.275 (0.210) |  |
| Persistently low-income x Female | **-1.231 (0.810)** |  | 0.748 (0.728) |  | -0.336 (0.214) |  |
| Female | 0.056 (0.373) |  | -1.255 (0.362) | *** | 0.526 (0.172) | ** |
|  |  |  |  |  |  |  |

*Note*. NW: not working; ST: standard daytime hours. Numbers represent unstandardized coefficients with standard errors in parentheses. All analyses were controlled for sociodemographic characteristics by age 22 and between ages 22 and 49, as detailed in the Measures section. **BOLDED** coefficients represent statistical significance at least at 5% level using the Wald test on the linear hypothesis about the joint significance of the interaction terms.

* *p* < .05, ** *p* < .01, *** *p* < .001.

Appendix Table 4. Regression Estimates of Work and Income Patterns Interacted with Race-Ethnicity on Health Outcomes at Age 50 (NLSY79)

|  | SF-12 Physical Function |  | SF-12 Mental Function |  | CES-D Depressive Symptoms |  |
| --- | --- | --- | --- | --- | --- | --- |
|  | b (sd) | sig. | b (sd) | sig. | b (sd) | sig. |
| Work schedule Patterns (ref: Mainly ST) |  |  |  |  |  |  |
| Mainly NW | -2.047 (0.790) | ** | -0.090 (0.664) |  | 0.294 (0.177) |  |
| ST to Evenings/Nights | -1.419 (0.638) | * | 0.470 (0.545) |  | 0.374 (0.169) | * |
| ST to Variable to ST | -0.803 (0.380) | * | -0.610 (0.358) |  | 0.288 (0.114) | ** |
| Mainly NW x Black | -1.056 (1.114) |  | -1.420 (0.953) |  | 0.134 (0.241) |  |
| Mainly NW x Hispanic | -0.796 (1.344) |  | -2.120 (1.213) |  | -0.240 (0.297) |  |
| ST to Evenings/Nights x Black | 0.403 (0.951) |  | -0.340 (0.747) |  | -0.121 (0.251) |  |
| ST to Evenings/Nights x Hispanic | -0.070 (1.190) |  | -0.998 (1.068) |  | -0.018 (0.364) |  |
| ST to Variable to ST x Black | -0.747 (0.694) |  | -0.086 (0.607) |  | -0.022 (0.197) |  |
| ST to Variable to ST x Hispanic | -0.250 (0.767) |  | 0.340 (0.642) |  | -0.228 (0.242) |  |
| Family income patterns (ref: upward to high-income) |  |  |  |  |  |  |
| Upward to middle-income | -1.343 (0.359) | *** | -0.201 (0.331) |  | 0.131 (0.130) |  |
| Persistently low-middle income | -3.556 (0.540) | *** | -1.371 (0.495) | ** | 0.750 (0.145) | *** |
| Persistently low-income | -6.978 (0.798) | *** | -3.712 (0.727) | *** | 1.029 (0.172) | *** |
| Upward to middle-income x Black | 0.060 (0.693) |  | -0.682 (0.590) |  | **0.127 (0.261)** |  |
| Upward to middle-income x Hispanic | -0.082 (0.697) |  | -0.448 (0.609) |  | **0.416 (0.306)** |  |
| Persistently low-middle income x Black | 1.146 (0.789) |  | 0.435 (0.695) |  | **-0.625 (0.261)** | ***** |
| Persistently low-middle income x Hispanic | 1.154 (0.839) |  | 0.734 (0.743) |  | **-0.030 (0.308)** |  |
| Persistently low-income x Black | 3.121 (1.004) | ** | 0.452 (0.894) |  | **-0.305 (0.262)** |  |
| Persistently low-income x Hispanic | 2.150 (1.165) |  | -0.088 (1.073) |  | **0.502 (0.315)** |  |
| Race-Ethnicity (ref: Non-Hispanic White) |  |  |  |  |  |  |
| Non-Hispanic Black (Black) | -0.099 (0.513) |  | 1.329 (0.453) | ** | -0.100 (0.223) |  |
| Hispanic | 0.370 (0.521) |  | 1.395 (0.481) | ** | -0.519 (0.259) | * |
|  |  |  |  |  |  |  |

*Note*. This analysis excludes those non-White, non-Black, and non-Hispanic due to the small sample size. NW: not working; ST: standard daytime hours. Numbers represent unstandardized coefficients with standard errors in parentheses. All analyses were controlled for sociodemographic characteristics by age 22 and between ages 22 and 49, as detailed in the Measures section. **BOLDED** coefficients represent statistical significance at least at 5% level using the Wald test on the linear hypothesis about the joint significance of the interaction terms.

* *p* < .05, ** *p* < .01, *** *p* < .001.

Appendix Table 5. Regression Estimates of Work and Income Patterns Interacted with Education on Health Outcomes at Age 50 (NLSY79)

|  | SF-12 Physical Function |  | SF-12 Mental Function |  | CES-D Depressive Symptoms |  |
| --- | --- | --- | --- | --- | --- | --- |
|  | b (sd) | sig. | b (sd) | sig. | b (sd) | sig. |
| Work schedule Patterns (ref: Mainly ST) |  |  |  |  |  |  |
| Mainly NW | -2.512 (0.828) | ** | -0.664 (0.692) |  | 0.442 (0.175) | ** |
| ST to Evenings/Nights | -1.650 (0.580) | ** | 0.434 (0.449) |  | 0.434 (0.159) | ** |
| ST to Variable to ST | -1.472 (0.452) | *** | -0.620 (0.396) |  | 0.414 (0.126) | *** |
| Mainly NW x Less than high school | **-1.424 (1.234)** |  | -1.678 (1.075) |  | -0.246 (0.245) |  |
| Mainly NW x Some college | **-0.268 (1.397)** |  | 0.026 (1.154) |  | -0.034 (0.303) |  |
| Mainly NW x College and plus | **4.179 (1.192)** | ******* | 1.147 (1.366) |  | -0.325 (0.495) |  |
| ST to Evenings/Nights x Less than high school | **0.294 (1.259)** |  | -0.582 (1.069) |  | -0.480 (0.287) |  |
| ST to Evenings/Nights x Some college | **0.981 (1.068)** |  | -0.838 (0.889) |  | -0.015 (0.309) |  |
| ST to Evenings/Nights x College and plus | **0.856 (1.483)** |  | 0.304 (0.993) |  | 0.003 (0.539) |  |
| ST to Variable to ST x Less than high school | **-0.119 (0.975)** |  | -0.395 (0.844) |  | -0.477 (0.218) | * |
| ST to Variable to ST x Some college | **1.056 (0.682)** |  | 0.419 (0.598) |  | -0.178 (0.215) |  |
| ST to Variable to ST x College and plus | **0.357 (0.703)** |  | -0.270 (0.740) |  | -0.086 (0.305) |  |
| Family income patterns (ref: upward to high-income) |  |  |  |  |  |  |
| Upward to middle-income | -1.389 (0.418) | *** | -0.740 (0.368) | * | 0.296 (0.159) |  |
| Persistently low-middle income | -3.234 (0.514) | *** | -1.333 (0.450) | ** | 0.694 (0.167) | *** |
| Persistently low-income | -5.183 (0.634) | *** | -4.060 (0.570) | *** | 1.223 (0.173) | *** |
| Upward to middle-income x Less than high school | 0.260 (1.319) |  | 0.770 (1.092) |  | -0.450 (0.326) |  |
| Upward to middle-income x Some college | -0.325 (0.655) |  | 0.670 (0.577) |  | -0.060 (0.252) |  |
| Upward to middle-income x College and plus | 0.948 (0.678) |  | 0.296 (0.679) |  | -0.152 (0.336) |  |
| Persistently low-middle income x Less than high school | 1.596 (1.310) |  | 0.817 (1.123) |  | -0.692 (0.316) | * |
| Persistently low-middle income x Some college | -0.363 (0.794) |  | 0.694 (0.723) |  | -0.109 (0.263) |  |
| Persistently low-middle income x College and plus | 0.226 (1.275) |  | 0.064 (1.126) |  | -0.089 (0.471) |  |
| Persistently low-income x Less than high school | 1.669 (1.347) |  | 1.629 (1.164) |  | -0.851 (0.308) | ** |
| Persistently low-income x Some college | -1.902 (1.280) |  | 0.077 (1.010) |  | -0.005 (0.288) |  |
| Persistently low-income x College and plus | -1.347 (5.061) |  | 4.480 (1.876) | * | 0.977 (0.751) |  |
| Race-Ethnicity (ref: High school degree) |  |  |  |  |  |  |
| Less than high school | -2.340 (1.134) | * | -1.467 (0.952) |  | 1.166 (0.284) | *** |
| Some college | -0.154 (0.476) |  | -0.404 (0.456) |  | 0.005 (0.210) |  |
| College and plus | 0.544 (0.476) |  | -0.180 (0.457) |  | -0.336 (0.237) |  |

*Note*. NW: not working; ST: standard daytime hours. Numbers represent unstandardized coefficients with standard errors in parentheses. All analyses were controlled for sociodemographic characteristics by age 22 and between ages 22 and 49, as detailed in the Measures section. **BOLDED** coefficients represent statistical significance at least at 5% level using the Wald test on the linear hypothesis about the joint significance of the interaction terms.

* *p* < .05, ** *p* < .01, *** *p* < .001.

Appendix Table 6. Predictive Margins of Work and Income Patterns by Gender on Health Outcomes at Age 50

|  | SF-12 Physical Function | SF-12 Mental Function | CES-D Depressive Symptoms |
| --- | --- | --- | --- |
| Male |  |  |  |
| Mainly NW | 47.33 [45.46, 49.20] | 53.04 [51.42, 54.66] | 0.19 [0.14, 0.24] |
| Mainly ST | 50.24 [49.78, 50.72] | 53.91 [53.50, 54.33] | 0.12 [0.11, 0.14] |
| ST to Evenings/Nights | 49.47 [48.51, 50.43] | 54.05 [53.29, 54.81] | 0.14 [0.10, 0.17] |
| ST to Variable to ST | 49.77 [49.11, 50.42] | 53.32 [52.72, 53.92] | 0.15 [0.12, 0.18] |
| Female |  |  |  |
| Mainly NW | 47.18 [46.04, 48.33] | 51.44 [50.44, 52.44] | 0.21 [0.17, 0.24] |
| Mainly ST | 49.86 [49.41, 50.32] | 52.56 [52.14, 52.98] | 0.17 [0.15, 0.18] |
| ST to Evenings/Nights | 47.96 [46.66, 49.27] | 52.76 [51.72, 53.80] | 0.24 [0.19, 0.29] |
| ST to Variable to ST | 48.05 [47.29, 48.81] | 51.93 [51.27, 52.60] | 0.20 [0.18, 0.23] |
| Male |  |  |  |
| Upward to middle-income | 50.68 [50.07, 51.29] | 54.52 [54.02, 55.02] | 0.11 [0.09, 0.13] |
| Upward to high-income | 51.51 [51.01, 52.00] | 54.65 [54.17, 55.13] | 0.09 [0.07, 0.10] |
| Persistently low-middle income | 48.16 [47.33, 48.98] | 53.79 [53.11, 54.47] | 0.16 [0.13, 0.19] |
| Persistently low-income | 46.96 [45.84, 48.07] | 50.60 [49.63, 51.57] | 0.24 [0.20, 0.29] |
| Female |  |  |  |
| Upward to middle-income | 49.17 [48.51, 49.83] | 52.63 [52.07, 53.19] | 0.17 [0.14, 0.19] |
| Upward to high-income | 51.16 [50.60, 51.71] | 53.37 [53.11, 54.47] | 0.14 [0.11, 0.16] |
| Persistently low-middle income | 48.35 [47.55, 49.14] | 52.18 [51.45, 52.90] | 0.20 [0.17, 0.23] |
| Persistently low-income | 45.37 [44.36, 46.39] | 50.07 [49.17, 50.96] | 0.28 [0.24, 0.32] |
|  |  |  |  |

*Note*. Predicted margins were based on the interaction analysis shown in Appendix Table 1. Numbers in brackets represent 95% confidence intervals. NW: not working; ST: standard daytime hours. Numbers represent unstandardized coefficients with standard errors in parentheses.

Appendix Table 7. Predictive Margins of Work and Income Patterns by Race-Ethnicity on Health Outcomes at Age 50

|  | SF-12 Physical Function | SF-12 Mental Function | CES-D Depressive Symptoms |
| --- | --- | --- | --- |
| Non-Hispanic White |  |  |  |
| Mainly NW | 47.35 [45.90, 48.81] | 52.42 [51.22, 53.62] | 0.21 [0.16, 0.26] |
| Mainly ST | 49.40 [48.91, 49.89] | 52.51 [52.05, 52.98] | 0.17 [0.15, 0.19] |
| ST to Evenings/Nights | 47.98 [46.79, 49.17] | 52.98 [51.98, 53.99] | 0.22 [0.18, 0.27] |
| ST to Variable to ST | 48.60 [47.90, 49.29] | 51.90 [51.26, 52.54] | 0.21 [0.18, 0.24] |
| Non-Hispanic Black |  |  |  |
| Mainly NW | 47.03 [45.44, 48.62] | 52.32 [50.93, 53.70] | 0.19 [0.14, 0.23] |
| Mainly ST | 50.14 [49.52, 50.75] | 53.82 [53.30, 54.35] | 0.13 [0.11, 0.15] |
| ST to Evenings/Nights | 49.12 [47.85, 50.39] | 53.95 [53.05, 54.86] | 0.16 [0.12, 0.21] |
| ST to Variable to ST | 48.59 [47.59, 49.58] | 53.13 [52.28, 53.97] | 0.17 [0.13, 0.20] |
| Hispanic |  |  |  |
| Mainly NW | 47.54 [45.37, 49.72] | 51.71 [49.70, 53.72] | 0.14 [0.10, 0.19] |
| Mainly ST | 50.39 [49.67, 51.11] | 53.92 [53.27, 54.56] | 0.14 [0.11, 0.17] |
| ST to Evenings/Nights | 48.90 [46.98, 50.82] | 53.39 [51.66, 55.12] | 0.18 [0.10, 0.26] |
| ST to Variable to ST | 49.34 [48.14, 50.54] | 53.65 [52.70, 54.60] | 0.14 [0.10, 0.19] |
| Non-Hispanic White |  |  |  |
| Upward to middle-income | 49.91 [49.31, 50.51] | 53.23 [52.73, 53.73] | 0.14 [0.12, 0.17] |
| Upward to high-income | 51.26 [50.75, 51.76] | 53.43 [52.94, 53.92] | 0.13 [0.11, 0.15] |
| Persistently low-middle income | 47.70 [46.77, 48.62] | 52.06 [51.22, 52.90] | 0.24 [0.20, 0.27] |
| Persistently low-income | 44.28 [42.84, 45.71] | 49.72 [48.41, 51.02] | 0.29 [0.24, 0.34] |
| Non-Hispanic Black |  |  |  |
| Upward to middle-income | 49.62 [48.70, 50.42] | 53.67 [52.94, 54.39] | 0.15 [0.11, 0.18] |
| Upward to high-income | 50.90 [50.07, 51.72] | 54.55 [53.83, 55.27] | 0.12 [0.08, 0.16] |
| Persistently low-middle income | 48.49 [47.56, 49.41] | 53.61 [52.85, 54.38] | 0.13 [0.10, 0.16] |
| Persistently low-income | 47.04 [45.99, 48.09] | 51.29 [50.41, 52.16] | 0.22 [0.18, 0.25] |
| Hispanic |  |  |  |
| Upward to middle-income | 50.04 [49.03, 51.06] | 53.93 [53.06, 54.79] | 0.12 [0.09, 0.16] |
| Upward to high-income | 51.47 [50.62, 52.32] | 54.58 [53.82, 55.34] | 0.08 [0.04, 0.11] |
| Persistently low-middle income | 49.07 [47.87, 50.26] | 53.94 [52.92, 54.95] | 0.14 [0.10, 0.18] |
| Persistently low-income | 46.64 [45.08, 48.21] | 50.78 [49.34, 52.22] | 0.27 [0.20, 0.33] |
|  |  |  |  |

*Note*. Predicted margins were based on the interaction analysis shown in Appendix Table 2. Numbers in brackets represent 95% confidence intervals. NW: not working; ST: standard daytime hours. Numbers represent unstandardized coefficients with standard errors in parentheses.

Appendix Table 8. Predictive Margins of Work and Income Patterns by Education on Health Outcomes at Age 50

|  | SF-12 Physical Function | SF-12 Mental Function | CES-D Depressive Symptoms |
| --- | --- | --- | --- |
| Less than high school |  |  |  |
| Mainly NW | 44.70 [42.82, 46.58] | 50.20 [48.55, 51.86] | 0.27 [0.20, 0.34] |
| Mainly ST | 48.64 [47.64, 49.64] | 52.55 [51.70, 53.39] | 0.24 [0.20, 0.28] |
| ST to Evenings/Nights | 47.28 [45.22, 49.34] | 52.40 [50.59, 54.21] | 0.23 [0.15, 0.31] |
| ST to Variable to ST | 47.04 [45.45, 48.64] | 51.53 [50.14, 52.92] | 0.23 [0.17, 0.28] |
| High school degree |  |  |  |
| Mainly NW | 47.75 [46.25, 49.24] | 52.66 [51.42, 53.90] | 0.20 [0.15, 0.24] |
| Mainly ST | 50.26 [49.79, 50.73] | 53.33 [52.89, 53.76] | 0.14 [0.12, 0.15] |
| ST to Evenings/Nights | 48.61 [47.56, 49.66] | 53.76 [52.99, 54.53] | 0.20 [0.15, 0.24] |
| ST to Variable to ST | 48.79 [48.02, 49.55] | 52.71 [52.04, 53.37] | 0.19 [0.16, 0.22] |
| Some college |  |  |  |
| Mainly NW | 46.81 [44.58, 49.03] | 52.63 [50.81, 54.45] | 0.19 [0.12, 0.25] |
| Mainly ST | 49.59 [48.92, 50.25] | 53.27 [52.67, 53.87] | 0.13 [0.11, 0.16] |
| ST to Evenings/Nights | 48.92 [47.25, 50.60] | 52.87 [51.43, 54.31] | 0.19 [0.12, 0.25] |
| ST to Variable to ST | 49.17 [48.25, 50.09] | 53.07 [52.29, 53.85] | 0.16 [0.13, 0.20] |
| College and plus |  |  |  |
| Mainly NW | 52.54 [49.92, 55.15] | 54.55 [52.10, 57.00] | 0.15 [0.04, 0.26] |
| Mainly ST | 50.87 [48.93, 52.81] | 54.07 [53.11, 55.03] | 0.13 [0.07, 0.20] |
| ST to Evenings/Nights | 50.08 [46.92, 53.23] | 54.80 [53.03, 56.58] | 0.18 [0.06, 0.31] |
| ST to Variable to ST | 49.75 [47.59, 51.92] | 53.18 [51.88, 54.47] | 0.17 [0.09, 0.25] |
| Less than high school |  |  |  |
| Upward to middle-income | 47.86 [46.52, 49.21] | 52.61 [51.54, 53.67] | 0.21 [0.16, 0.26] |
| Upward to high-income | 48.99 [46.92, 51.07] | 52.58 [50.82, 54.33] | 0.23 [0.15, 0.31] |
| Persistently low-middle income | 47.35 [46.15, 48.56] | 52.06 [50.99, 53.13] | 0.23 [0.18, 0.28] |
| Persistently low-income | 45.48 [44.29, 46.67] | 50.14 [49.10, 51.19] | 0.30 [0.25, 0.35] |
| High school degree |  |  |  |
| Upward to middle-income | 50.10 [49.49, 50.71] | 53.64 [53.14, 54.14] | 0.14 [0.11, 0.16] |
| Upward to high-income | 51.49 [50.89, 52.09] | 54.38 [53.83, 54.93] | 0.11 [0.08, 0.13] |
| Persistently low-middle income | 48.26 [47.45, 49.06] | 53.04 [52.36, 53.73] | 0.19 [0.16, 0.22] |
| Persistently low-income | 46.31 [45.28, 47.33] | 50.32 [49.38, 51.26] | 0.28 [0.24, 0.32] |
| Some college |  |  |  |
| Upward to middle-income | 49.94 [49.08, 50.80] | 53.93 [53.24, 54.62] | 0.12 [0.09, 0.16] |
| Upward to high-income | 51.66 [51.03, 52.29] | 54.00 [53.58, 54.62] | 0.10 [0.08, 0.13] |
| Persistently low-middle income | 48.06 [46.96, 49.16] | 53.36 [52.36, 54.36] | 0.17 [0.12, 0.21] |
| Persistently low-income | 44.57 [42.44, 46.71] | 50.02 [48.22, 51.81] | 0.27 [0.19, 0.34] |
| College and plus |  |  |  |
| Upward to middle-income | 52.22 [51.14, 53.29] | 53.84 [52.80, 54.88] | 0.08 [0.04, 0.12] |
| Upward to high-income | 52.66 [52.00, 53.32] | 54.29 [53.61, 54.97] | 0.07 [0.05, 0.10] |
| Persistently low-middle income | 49.65 [47.38, 51.92] | 53.02 [51.04, 55.00] | 0.13 [0.04, 0.21] |
| Persistently low-income | 46.13 [36.28, 55.98] | 54.71 [51.21, 58.20] | 0.40 [0.08, 0.73] |
|  |  |  |  |

*Note*. Predicted margins were based on the interaction analysis shown in Appendix Table 3. Numbers in brackets represent 95% confidence intervals. NW: not working; ST: standard daytime hours. Numbers represent unstandardized coefficients with standard errors in parentheses.

Appendix Figure 1. Sequence Cluster Solutions on Work Between Ages 22-49, NLSY79=7,871


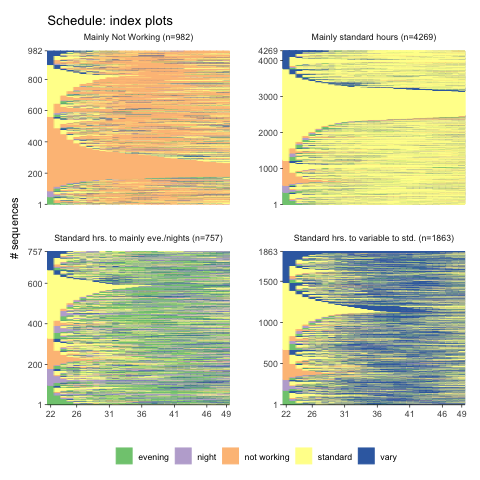


Appendix Figure 2. Sequence Cluster Solutions on Family Income Between Ages 22-49, NLSY79=7,871


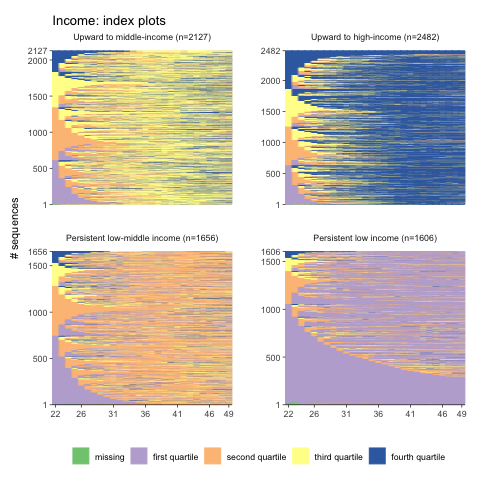

Supplement: Supplementary file 1 — Supplementary Material 1: Appendix A. Empirical Strategies. Appendix B. Clustering Methodology. Appendix Fig. 1. Sequence Cluster Solutions on Work Between Ages 22–49, NLSY79 = 7,871. Appendix Fig. 2. Sequence Cluster Solutions on Family Income Between Ages 22–49, NLSY79 = 7,871. Appendix Table 1. Descriptive Statistics of Analyzed Variables by Work Patterns, NLSY79 (N = 6,672). Appendix Table 2. Descriptive Statistics of Analyzed Variables by Family Income Patterns, NLSY79 (N = 6,672). Appendix Table 3. Regression Estimates of Work and Income Patterns Interacted with Gender on Health Outcomes at Age 50 (NLSY79). Appendix Table 4. Regression Estimates of Work and Income Patterns Interacted with Race-Ethnicity on Health Outcomes at Age 50 (NLSY79). Appendix Table 5. Regression Estimates of Work and Income Patterns Interacted with Education on Health Outcomes at Age 50 (NLSY79). Appendix Table 6. Predictive Margins of Work and Income Patterns by Gender on Health Outcomes at Age 50. Appendix Table 7. Predictive Margins of Work and Income Patterns by Race-Ethnicity on Health Outcomes at Age 50. Appendix Table 8. Predictive Margins of Work and Income Patterns by Education on Health Outcomes at Age 50. [file 12889_2025_23320_MOESM1_ESM.docx]
